# ANKRD1 Promotes Breast Cancer Metastasis by Activating NF-*κ*B-MAGE-A6 Pathway

**DOI:** 10.3390/cancers16193306

**Published:** 2024-09-27

**Authors:** Penchatr Diskul-Na-Ayudthaya, Seon Joo Bae, Yun-Ui Bae, Ngu Trinh Van, Wootae Kim, Seongho Ryu

**Affiliations:** 1 Soonchunhyang Institute of Medi-bio Science (SIMS), Department of Integrated Biomedical Sciences, Soonchunhyang University, Asan-si 31151, Republic of Korea; penchatr@cri.or.th (P.D.-N.-A.); bsj@sch.ac.kr (S.J.B.); tvngu@hcmus.edu.vn (N.T.V.); 2 Laboratory of Biochemistry, Chulabhorn Research Institute, Bangkok 10210, Thailand; 3 Precision Medicine Lung Cancer Center, Konkuk University Medical Center, Konkuk University, Seoul 05030, Republic of Korea; 2022c010@kuh.ac.kr; 4 Department of Pathology, College of Medicine, Soonchunhyang University, Asan-si 311151, Republic of Korea

**Keywords:** ANKRD1, MAGE-A6, NF-κB pathway, metastasis, breast cancer

## Abstract

**Simple Summary:**

Change in protein expression is the key driving force for tumorigenesis. Increased expression of Ankyrin Repeat Domain 1 (ANKRD1) acts as a co-activator of the p53 tumor suppressor protein and is associated with cancer drug resistance and poor survival of cancer patients. In this study, we found increased levels of ANKRD1 in highly metastatic breast cancer cell lines and human tissues of high-grade breast cancer. We also demonstrated that ANKRD1 is upstream of NF-κB-MAGE-A6 and plays a role in cancer metastasis. Our results show that the expression of ANKRD1 has significant clinical diagnostic significance in breast cancer metastasis.

**Abstract:**

Early detection and surgical excision of tumors have helped improve the survival rate of patients with breast cancer. However, patients with metastatic cancer typically have a poor prognosis. In this study, we propose that ANKRD1 promotes metastasis of breast cancer. ANKRD1 was found to be highly expressed in the MDA-MB-231 and MDA-LM-2 highly metastatic breast cancer cell lines compared to the non-metastatic breast cancer cell lines (MCF-7, ZR-75-30, T47D) and normal breast cancer cells (MCF-10A). Furthermore, high-grade tumors showed increased levels of ANKRD1 compared to low-grade tumors. Both in vitro and in vivo functional studies demonstrated the essential role of ANKRD1 in cancer cell migration and invasion. The previous studies have suggested a significant role of NF-κB and MAGE-A6 in breast cancer metastasis, but the upstream regulators of this axis are not well characterized. Our study suggests that ANKRD1 promotes metastasis of breast cancer by activating NF-κB as well as MAGE-A6 signaling. Our findings show that ANKRD1 is a potential therapeutic target and a diagnostic marker for breast cancer metastasis.

## 1. Introduction

Breast cancer (BRCA) is one of the most common cancers in women [1]. The incidence of breast cancer and the associated mortality has shown a progressive increase over the years. Breast cancer accounts for approximately 25% of all new cancer cases and approximately 15% of all cancer-related deaths [2]. Metastasis is the most serious problem of breast cancer, and the estimated survival rate of patients after diagnosis of metastatic cancer is 10–20% [3,4,5,6,7,8,9]. Furthermore, the molecular mechanisms involved in the metastasis of breast cancer are poorly understood. Metastatic tumors are often diagnosed at late stages when the primary tumor has already started metastasizing and has become large in size [10]. However, there are no accurate prognostic predictions and treatments for breast cancer metastases. Therefore, it is urgent to identify the candidate proteins that might be therapeutic targets in breast cancer patients with metastatic disease [11,12,13,14,15,16].

The main reason for the failure of breast cancer treatment is metastasis, which is a multi-step process involving the alteration and modification of complex molecular pathways [17]. Cancer cells typically demonstrate altered morphology during metastasis and begin to lose the ability to attach to the neighboring cells and the basement membrane. These cells then undergo EMT and enter the systemic circulation, disseminate to distant organs and adapt to grow [17,18]. Invasion and metastasis are the hallmarks of cancer [19], which can be triggered by many factors, such as epigenetic factors, soluble signals, cell-cell interactions, adhesive signals from extracellular matrix (ECM) components and ECM mechanical pressure [20]. The switch from an epithelial to mesenchymal phenotype is usually triggered by complex networks involved in gene transcriptional control, such as E-Cadherin, N-Cadherin, fibronectin, vimentin, SNAIL2, twist, ZEB1, and ZEB2 [21]. Understanding the underlying molecular mechanisms of the EMT can help increase the survival rates of breast cancer patients.

In previous work, we screened the differential gene expression between non-metastatic and highly metastatic breast cancer cell lines by using cDNA microarray and found that ANKRD1 was upregulated in the highly metastatic cell lines (unpublished data). Two reports consistently demonstrated that Ankyrin repeat domain 1 (ANKRD1), also known as cardiac ankyrin repeat protein (CARP), was an EMT-associated-gene [22,23]. Since EMT is crucial for invasion and metastasis, it is interesting to explore the role of ANKRD1 in cancer invasion. ANKRD1 belongs to the muscle ankyrin repeat protein (MARP) family. It is known to be functionally involved in transcriptional regulation, mechano-sensing, and sarcomere in the heart [24,25]. ANKRD1 is one of the downstream components of the hippo pathway [26,27]. It is involved in many pathways: (a) downstream of YAB in the regulation of gene expression of hepatic stellate cell activation [28,29]; (b) negative transcriptional co-factor of Y-box binding protein (YB-1) protein in cardiomyogenesis [30]; (c) negative transcriptional regulator of nuclear factor k-light-chain-enhancer (NF-κB) in the regulation of the tumor suppressor p53; (d) acting as a co-transcription factor with GATA-4 in the regulation of stress response and anti-apoptosis [30,31]. Furthermore, ANKRD1 is known to be related to several signaling core molecules such as four-and-a-half LIM domains (FHL2), calsequestrin 2 (CASQ2), 14-3-3 proteins, and protein kinase C alpha (PKCα) [29,32,33,34,35,36,37,38,39,40,41]. ANKRD1 is also involved in various processes, including hypertrophic stress responses [42], cardiac fibrosis, deposition of ECM [43,44], apoptotic cell injury, hypoxia, and cardiomyocyte apoptosis [45,46,47,48,49]. In addition, a recent study demonstrated that ANKRD1 enhances platinum resistance in colorectal cancer and decreases cisplatin sensitivity in ovarian cancer [48,49].

In this study, we aimed to investigate the role of ANKRD1 in breast cancer metastasis. We studied the downstream pathway of ANKRD1 and found that ANKRD1 promotes breast cancer cell migration and metastasis through the NF-κB and MAGE-A6 pathway.

## 2. Materials and Methods

### 2.1. Cancer Cells and Cell Culture

Human breast cancer cell lines, MCF-10A, MCF7, T47D, and MDA-MB-231, were purchased from the ATCC. The MDA-MB-231-derived lung metastatic cell line MDA-MB-231-LM-2 (LM-2) was a generous gift from Dr. Joan Massague (Sloan Kettering Institute, New York, NY, USA). MCF-10A was grown in DMEM-F12 (Invitrogen, Waltham, MA, USA) supplemented with 2% horse serum (Invitrogen), 0.5 µg/mL of hydrocortisone (Sigma, St. Louis, MO, USA), cholera toxin (List Biological Laboratories, Campbell, CA, USA) 100 ng/mL, insulin (Sigma), and 1% penicillin/streptomycin (Corning, NY, USA). ZR-75-30 and T47D cell lines were grown in RPMI-1640 (Corning) supplemented with 10% fetal bovine serum (Corning) and 1% penicillin/streptomycin. MDA-MB-231 and LM-2 cells were cultured in DMEM medium (Corning) containing 10% fetal bovine serum and 1% of penicillin–streptomycin solution. All cell lines were maintained in a humidified incubator with 5% CO_2_ at 37 °C.

### 2.2. RNA Extraction and Real-Time PCR

Total RNA was extracted using the RNeasy mini kit (Qiagen, Hilden, Germany). cDNA synthesis was performed using the iScript cDNA synthesis kit (Bio-Rad, Hercules, CA, USA). Quantitative real-time PCR was performed using IQ SYBR green supermix (Bio-Rad). The primers used for real-time PCR are listed in Table S1. All experiments were performed in triplicate, and all genes were normalized to *Gapdh* gene levels as the loading control.

### 2.3. Protein Extraction and Western Blot Analysis

Protein was extracted using RIPA lysis buffer (Merck Millipore, Burlington, MA, USA) containing complete™ Protease Inhibitor Cocktail (Sigma-Aldrich, St. Louis, MO, USA) and phosphatase inhibitor cocktail 2 (Sigma-Aldrich). Protein was collected by high-speed centrifugation and its concentration was measured using the PierceTM BCA protein assay kit (Thermo Scientific^TM^, Waltham, MA, USA), after which Western blotting was performed. The primary antibodies used were anti-CARP (PA5-30538, Thermo Fisher Scientific), anti-Akt (9272, Cell Signaling Technology, Danvers, MA, USA), anti-pAKT (9271, Cell Signaling Technology), anti-NF-κB (3034, Cell Signaling Technology), anti-pNF-κB (3031, Cell Signaling Technology), anti-IκKα (4812, Cell Signaling Technology), anti-pIκKα (2859, Cell Signaling Technology), anti-MAGE-A6 (ab38495, Abcam, Cambridge, UK), anti-E-CADHERIN (3195S, Cell Signaling Technology), anti-N-CADHERIN (4061S, Cell Signaling Technology), anti-VIMENTIN (5741S, Cell Signaling Technology), and anti-TUBULIN (2148, Cell Signaling Technology). After overnight incubation with primary antibody at 4 °C, the membranes were incubated with secondary antibodies conjugated with horseradish peroxidase (1:5000, Invitrogen). ECL prime Western blotting reagents (GE Healthcare, Wien, Austria) were used to visualize protein bands (ChemiDoc™ touch image system, Bio-Rad).

### 2.4. Plasmids, shRNA Lentivirus and siRNA Transfection

ANKRD1 human tagged ORF clone (RC205609L4) and planti-C-mGFP-p2A-PURO (PS100093) were purchased from OriGENE (ORIGENE, MD, USA). ANKRD1 shRNA RNA lentiviruses and plati-puro-GFP-pLKO.1 control lentivirus were purchased from Sigma-Aldrich (TRCN0000149714, TRCN0000412849, and TCRN0000072182). All plasmids were transformed into DH5α competent cells. DNA was extracted using Endotoxin-free plasmid DNA purification (Macherey-Nagel, Duren, Germany). Transfection was performed using Lipofector-pMax (Aptabio, Yongin-si, Republic of Korea) according to the manufacturer’s protocol. Lentivirus was transformed using polybrene (Merck Millipore) at 5 µg/mL. The siRNAs of MAGE-A6 and negative control (SN1001) were purchased from Bioneer (Seoul, Republic of Korea). The siRNA of NF-κB was purchased from Genolution (Seoul, Republic of Korea) (Table S2). Cells were seeded until 70% confluence and then transfected in Opti-MEMTM (Gibco) using Lipofectamine RNAiMAX (Invitrogen) for 6 h. Then, cells were incubated in completed media and further incubated for 72 h.

### 2.5. Cell Proliferation Assay

Cells were labeled with the CellTrace™ CFSE cell proliferation kit (Thermo Fisher Scientific) according to the manufacturer’s protocol. Then, cells were counted for 7 days by flow cytometry using FACS Diva software V.6.1.3 (FACSCANTO II, BD Biosciences, Milpitas, CA, USA). Cell proliferation was calculated using FlowJo software V.10.10. Cell proliferation was also analyzed using EZ-Cytox cell viability assay kit (EZ-3000, DoGENBio, Seoul, Republic of Korea), according to the manufacturer’s instructions.

### 2.6. In Vitro Wound Healing, Transwell Migration and Invasion Assay

For wound healing assays, cells (1 × 10^5^ cell/well) were cultured to 90% confluence, scraped with sterile pipette tips, and washed twice with PBS before adding media. After 12–72 h of incubation in a CO_2_ incubator, photographs of migrated cells were obtained and quantified using ImageJS. For transwell migration assays, 1 × 10^5^–2 × 10^5^ cells/well were seeded in the upper chamber in a serum-free medium, while a medium with serum was added to the lower chamber. After incubating for 12–72 h, cells were fixed in 4% paraformaldehyde (SM-P01-100, GeneAll, Seoul, Republic of Korea) and 25% methanol and stained with 0.5% crystal violet. Photographs of migrated cells were obtained, and the number of migrating cells was eluted in elution buffer (1N HCL and MeOH). For cell invasion, the upper chamber of transwells was coated with 10% Matrigel (3566230, Corning), then cells were seeded and incubated for 48–72 h. Samples were fixed and stained according to the migration protocol. To confirm the effect of MAGE-A6 on cell wound healing, migration, and invasion, cells were seeded as mentioned above and incubated with 3 ng/mL of recombinant MAGE-A6 (ab126656, Abcam) after scratching the cells for wound healing assay and added in the upper chamber for migration and invasion. Then, cells were incubated for another 12–24 h.

### 2.7. In Vivo Imaging and Mouse Pulmonary Metastasis Model

All animal experiments were conducted in accordance with the protocol approved by the Institutional Animal Care and Use Committee at the Soonchunhyang University (protocol no. 2017-0039). For orthotopic injection, 1 × 10^6^ cells per 100 μL in sterile phosphate-buffer saline (PBS) of LM-2 control vector and ANKRD1-knockdown cell lines (containing GFP) were orthotopically injected into the mammary fat pad of 6–8-week-old immunodeficient NOD/SCID mice (n = 10 per each group), which were purchased from the Jackson laboratory. Images of the GFP signal were detected every week using IVIS**^®^** Lumina XEMS series III (PerkinElmer, Waltham, MA, USA). After 3 weeks, primary tumors were removed from mice, and the tumor volume was measured. Then, mice were maintained for another 1 week. All mice were sacrificed, and GFP signals were detected in all organs, which were then fixed in 4% paraformaldehyde solution (Thermo Fisher Scientific); organ metastasis was quantified using IVIS software V.4.8.2. For the metastasis assay, 1 × 10^6^ cells were injected through the tail vein with LM-2 control vector and LM-2-ANKRD1-knockdown cells, which was followed by the injection of the luciferin substrate (Promega). Luciferase signals were detected using IVIS**^®^** Lumina XEMS series III. Then, luciferase signals were detected weekly. After 4 weeks, mice were sacrificed, and organs were fixed in 4% paraformaldehyde solution (Thermo Fisher Scientific). All experiments were performed in triplicate.

### 2.8. Immunohistochemistry (IHC)

A human breast cancer tissue array containing 100 cancer cases of different grades was purchased (BR10011, US Biomax, Rockville, MD, USA), and mouse organs were collected and maintained in 4% paraformaldehyde solution and used to detect ANKRD1 expression. Briefly, the slides were incubated overnight with anti-ANKRD1 antibody at 4 °C and subsequently with the VECTASTAIN**^®^** ABC kit (Vector Laboratories, Newark, CA USA), followed by the DEB-HRP substrate kit (Vector Laboratories). IHC results were analyzed as follows: firstly, the staining intensity was scored by pathologists, who were blinded to the clinical samples by incorporating both staining intensity and a percentage of stained cells at each intensity level. The intensity score was classified into three groups as follows: ‘+’ for weak staining; ‘++’ for moderate staining and ‘+++’ for strong staining. Secondly, a semi-quantitative analysis of the immunohistochemistry images was performed using ImageJ software V 1.54. Color deconvolution analysis was carried out using the IHC profiling plugin. A DAB image that had been deconvoluted was then used to calculate the staining intensity. The staining intensity was measured using the “mean grey value” metric. The average staining intensities were calculated for each sample for each measurement of the total tissue area of vision. Pixel intensity values in ImageJ range from 0 to 255, where 0 indicates the darkest shade of color and 255 indicates the lightest. Since 255 is the highest intensity value of an RGB image that can be analyzed in ImageJ, the intensity of a stained area of interest (ROI) was deducted from that value. The relative stained area to the total tissue area was used to calculate the stained tissue. An ANOVA analysis was used to analyze the ANKRD1 staining score of different grades of tissue samples. Significant *p*-values are presented as ** < 0.001 and * < 0.05.

### 2.9. Hematoxylin and Eosin (H&E) Staining

The staining protocol, briefly, sample slides were deparaffinized in xylene (Duksan, Seoul, Republic of Korea) and hydrated by passage through a series of 100%, 95%, 90%, 80%, 70%, and 50% ethanol (Samchun, Seoul, Republic of Korea). Slides were then washed with tap water, followed by staining with hematoxylin (YD Diagnostics, Yongin-si, Republic of Korea). Slides were then rinsed with tap water, stained with eosin (BBC, Washington, WA, USA), dipped in 1% acetic acid (Sigma-Aldrich) in ethanol, and rinsed with tap water. Next, samples were dehydrated by passage through xylene and graded ethanol series (50%, 70%, 80%, 90%, 95%, and 100%). Lastly, Permount™ mounting medium (Fisher Scientific) was dropped over the coverslip and placed on top of the sample slides.

### 2.10. RNA-SEQ

RNA extraction from LM-2 control vector and LM-2-knockdown cell lines was performed as previously described in this manuscript. All samples were sent to Macrogen (Seoul, Republic of Korea) for construction of a library and further RNA-SEQ analysis. Analyzed data were provided by Macrogen. Samples cut-offs were adjusted to a *p*-value < 0.05. Significant *p*-values are presented as *** < 0.001, ** < 0.01 and * < 0.05.

### 2.11. Statistical Analysis

All variables and mean (±SD) values from three independent experiments are presented. Differences between two treatment groups were assessed using the study *t*-test, and differences more than two treatment groups were assessed using an ANOVA with the GraphPad Prism program V. 10 (Graphpad Software Inc., San Diego, CA, USA). Significant *p*-values are presented as ** < 0.001 and * < 0.05.

## 3. Results

### 3.1. ANKRD1 Is Upregulated in Highly Metastatic Breast Cancer Cell Lines

**Figure 1:**
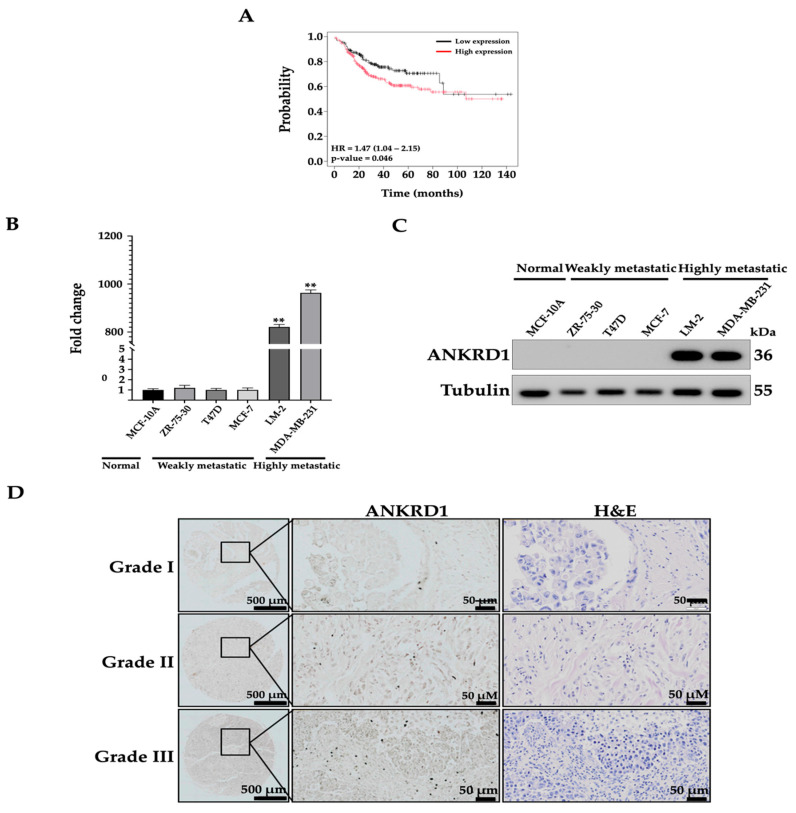
High ANKRD1 expression correlates with poor prognosis, aggressiveness and tumor progression of breast cancer. (**A**) Kaplan–Meier survival analysis of distant metastasis-free survival (DMFS) breast cancer patients with metastatic disease based on ANKRD1 expression using mRNA gene chip (HR; the hazard ratio) (https://kmplot.com). The following parameters were set: (1) lymph node status: positive; (2) probe set: only JetSet best probe; (3) grade: 3; and (4) survival: DMFS. Patients with higher ANKRD1 expression showed lower survival rates compared to those with low expression of ANKRD1. (**B**,**C**) Real-time PCR and Western blot results showing ANKRD1 fold change in normal breast (MCF-10A) and non-metastatic (ZR-75-30, T47D and MCF-7) and highly metastatic breast cancer cells (LM-2 and MDA-MB-231). (**D**) IHC staining (left panel) of ANKRD1 in human breast tissue array showing progressively higher ANKRD1 expression depending on the grade of breast cancer tissue. The right panel shows H&E-stained sections.

Based on ANKRD1 expression using an mRNA gene chip, we calculated the survival rate of patients with distant metastasis-free survival (DMFS) breast cancer using Kaplan–Meier survival analysis (https://kmplot.com, accessed on 17 September 2024). A total of 429 patients with breast cancer were included in this data collection, and the following parameters were specified: (1) survival: DMFS; (2) probe set: only JetSet best probe; (3) lymph node status: positive; and (4) grade: 3. Patients with higher expression levels of ANKRD1 had a lower survival rate when compared to those with lower expression levels of ANKRD1 (Figure 1A). However, the association between low survival rates and high expression of ANKRD1 does not correlate with any specific subtype of breast cancer (Figure S1A–F). In vitro studies revealed significant upregulation of ANKRD1 at both the transcriptional and translational level in highly metastatic cancer cells (Figure 1B,C). To confirm whether ANKRD1 is highly expressed in highly metastatic cells compared to non-metastatic cells, we performed immunohistochemical staining (IHC) of ANKRD1 in the breast cancer tissue array. The criteria for the grading system are based on several factors. Grade 1 refers to cancer cells that grow slowly and resemble normal breast tissue (non-aggressive). Grade 2 indicates that the cells grow more quickly and have characteristics intermediate between grades 1 and 3 (moderately aggressive). Grade 3 refers to cancer cells that look very different from normal cells, grow rapidly, spread quickly, and are more likely to metastasize to secondary organs (highly aggressive) [50]. The IHC results demonstrated that ANKRD1 expression was grade dependent, with higher signal levels detected at higher grades (Figure 1D and Table 1). Collectively, these results demonstrated that ANKRD1 expression level correlated with higher grade and aggressiveness of breast cancer and suggested that ANKRD1 may play a significant role in breast cancer progression and metastasis.

### 3.2. ANKRD1 Induces Cell Migration in Weakly Metastatic Breast Cancer Cells

**Figure 2:**
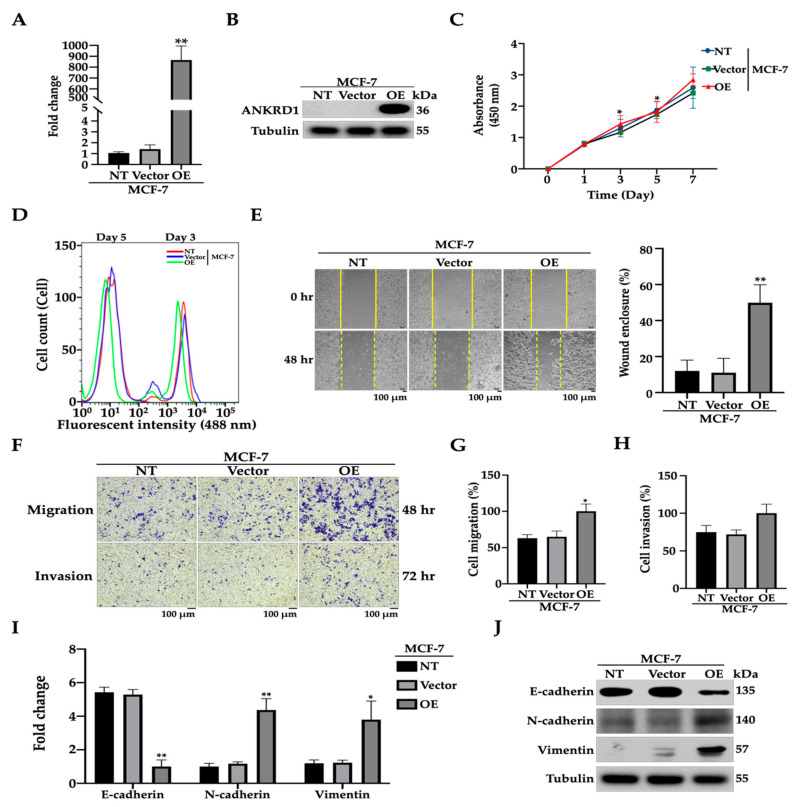
Overexpression of ANKRD1 enhances migration of weakly metastatic MCF-7 cells. (**A**,**B**) Results of real-time PCR and Western blot assay showing the gene and protein expression in the ANKRD1-overexpressed cell lines (MCF-7-ANKRD1 OE, OE). (**C**) Cell proliferation of ANKRD1-overexpressing cells compared to vector and normal MCF-7 was determined by EZ-Cytox assay and (**D**) CellTrace™ CFSE Cell proliferation kit; cell numbers were counted by flow cytometry using FACS Div and calculated using FlowJo software. (**E**) Representative images depicting wound healing quantified as a percentage of the healed wound area and analyzed using Image JS. (**F**–**H**) Migration and invasion assays performed with MCF-7, vector and OE. OE cells showed increased wound healing, migration, and invasion compared to vector and NT cells (**I**,**J**) Real-time PCR and Western blot showing the gene and protein expression involved in EMT. OE cells showed reduced expression of E-cadherin and increased expression of N-cadherin and vimentin compared to vector. NT: MCF-7 parental; Vector: MCF-7-emtpy vector; OE: MCF-7-ANKRD1 overexpression. All experiments were performed in triplicate.

To determine the involvement of ANKRD1 in the migration of breast cancer cells, we established stable MCF7-ANKRD1-overexpressing cell lines (OE) by transfecting lentivirus vector into non-metastatic breast cancer cells (MCF-7). MCF7-ANKRD1 OE cells showed the overexpression of ANKRD1 at both the transcriptional and translational level (Figure 2A,B). Functional phenotypic experiments showed no difference in the proliferation rates of stable MCF7-ANKRD1 OE cells, the empty vector (control) cells, and parental cells, using two different methods (Figure 2C,D). Wound healing and migration assays showed that the upregulation of ANKRD1 enhanced wound healing and cell migration. However, MCF7-ANKRD1 OE cells showed no significant difference in terms of cell invasion (Figure 2E–H). Similar results were observed in T47D-ANKRD1 OE cells (Figure S2A,B). Moreover, there was no significant difference in proliferation rates between T47D-ANKRD1 OE cells, the control cells, and parental cells (Figure S3C), whereas enhanced cell wound healing was observed (Figure S3D). These results indicate that ANKRD1 may regulate only cell migration without increasing cell proliferation and cell invasion in breast cancer.

We investigated whether the underlying mechanism(s) of ANKRD1-mediated breast cancer migration is correlated with epithelial mesenchymal transition (EMT), migration, adhesion and cancer-associated fibroblasts (CAFs). The results showed the upregulation of N-cadherin, vimentin, fibroblast activation protein-α (FAP), Periostin (POSTN), Tenascin C (TNC), and Myosin light chain kinase (MYLK) in both MCF7-ANKRD1 OE and T47D-ANKRD1 OE cells. However, E-cadherin was reduced in MCF7-ANKRD1 OE and T47D-ANKRD1 OE cells (Figure 2I,J, Figures S2A,B and S3E–G).

### 3.3. Knockdown of ANKRD1 Suppresses Migration and Invasion of Highly Metastatic LM-2 Cells

**Figure 3:**
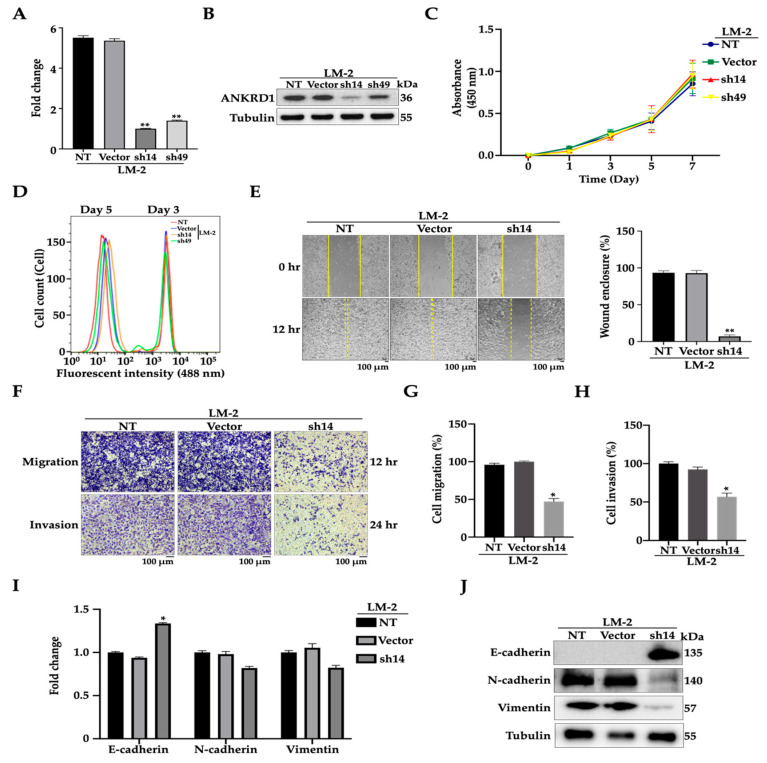
Knockdown of ANKRD1 suppresses migration and invasion of highly metastatic LM-2 cells. (**A**,**B**) Real-time PCR and Western blots showing gene and protein expression of ANKRD1-knockdown cell lines (sh14 and sh49). (**C**) Cell proliferation of LM-2-ANKRD1 knockdown cells compared to vector and LM-2 was determined by EZ-Cytox assay and (**D**) by CellTrace™ CFSE Cell proliferation kit; cell numbers were counted by flow cytometry using FACS Diva and calculated using FlowJo software. Representative images depicting (**E**) wound healing (**F**–**H**), migration, and invasion assays performed with NT, Vector, and sh14. sh14 cells showed decrease in wound healing, migration, and invasion compared to vector and NT cells. (**I**,**J**) Real-time PCR and Western blots showing the expression of EMT markers. Sh14 cells showed increased expression of E-cadherin and decreased expression of N-cadherin and vimentin compared to vector and NT. NT: LM-2 parental; Vector: LM-2 with empty vector; sh14: LM-2-ANKRD1 knockdown with sh14 and sh49: LM-2-ANKRD1 knockdown with sh49. All experiments were performed in triplicate.

To confirm the previous results of ANKRD1 overexpression, we established stable MDA-MB-LM2-ANKRD1 knockdown cell lines using the lentivirus vector. Virus particles were transfected into highly metastatic breast cancer cells (LM2). We first confirmed the reduction of ANKRD1 at both the transcriptional and translational levels (Figure 3A,B). There were no significant differences in the cell proliferation rates between stable LM2-ANKRD1 knockdown cells (sh14 and sh49) and the empty vector (control) as well as parental cells (Figure 3C,D). However, the expression of ANKRD1 in sh14 was lower than sh49, so we used sh14 for further experiments. The stable LM2-ANKRD1 knockdown cell line (sh14) showed a significant decrease in wound healing, migration, and invasion ability compared to the control vector and parental cells (Figure 3E–H), which was consistent with our previous overexpression results. In addition, mRNA and protein expression levels of migration, adhesion, and CAF markers showed no significant difference, except for the EMT markers. E-CAD was increased in the LM2-ANKRD1 knockdown cell line, while the protein expressions of N-CAD and VIM were reduced (Figure 3I,J and Figure S4A,B). These in vitro results suggested that ANKRD1 may trigger breast cancer migration through EMT.

### 3.4. ANKRD1 Knockdown Suppresses Tumorigenesis and Metastasis

**Figure 4:**
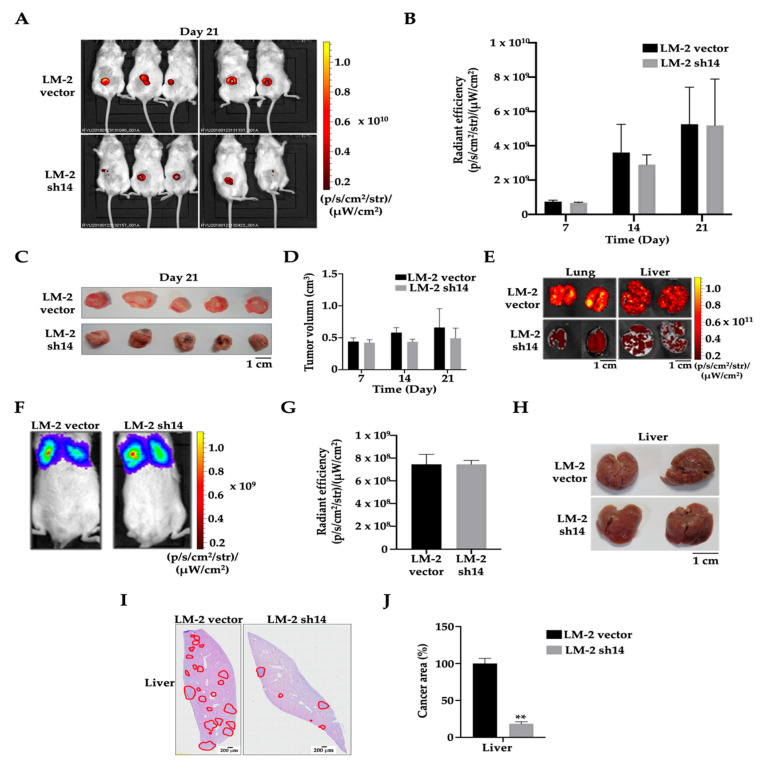
ANKRD1 knockdown reduces metastasis efficiency of breast cancer cells in two in vivo metastasis models. (**A**,**B**) Representative bioluminescence (BLI) images of animals showing primary tumors (PT) at day 21, derived from orthotopic injections of LM-2 vector control (vector) and LM-2 ANKRD1 knockdown (LM-2 sh14) cells into the mammary fat pad. On day 21 after orthotopic injection into the mammary fat pad, tumor growth at the injection site and GFP was detected by IVIS and quantified based on radiant efficiency, comparing vector and LM-2 sh14 groups (day 21, n = 10 per group). (**C**) Comparison of the size of primary tumors from vector and LM-2 sh14 groups after removing the primary tumor. (**D**) Quantitation of tumor volume (cm^3^) comparing control and LM-2 sh14 groups. The results show no significant change in tumor volume and tumorigenesis in the LM-2 sh14 cell group compared to the vector. Data presented as mean ± SD. (**E**) Radiant efficiency of the lung and liver comparing vector and LM-2 sh14 groups and ex vivo GFP signals, detected using IVIS. (**F**,**G**) Representative BLI images of animals on the first day of tail vein injection and quantitation of radiant efficiency in mice (day 1, n = 10 per group). (**H**) Liver metastasis with control and LM-2 sh14 cells. The results show increased metastasis of cancer cells in control compared to LM-2 sh14 group. (**I**,**J**) H&E staining of liver tissue from tail-vein-injected mice; red circles show the cancerous lesions in the tissues, which are increased in the control group compared to the LM-2 sh14 group. LM-2 Vector (control): LM-2 with empty vector; LM-2 sh14: LM-2-ANKRD1 knockdown with sh14. Ten NOD.SCID mice were used for each set, and all experiments were performed in triplicate.

Since we found that ANKRD1 contributes to cancer cell migration and invasion, we further investigated its role by performing in vivo mouse experiments. LM2-ANKRD1 knockdown cells (sh14) or control cells (vector) were orthotopically injected into NOD/SCID mice. After 3 weeks, primary tumors were removed from mice, and the results showed that tumor volume and tumorigenesis in LM-2-ANKRD1 knockdown cells did not differ significantly from the control (Figure 4A–D). To evaluate the metastatic potential of the cancer to metastasize, mice were maintained for another week and then sacrificed. GFP signals were studied in all organs, with the results showing that mice injected with LM2-ANKRD1 knockdown cells had significantly reduced lung and liver metastasis, compared to mice injected with control cells (Figure 4E and Figure S5A,B). These results indicate that when ANKRD1 is silenced in LM2-ANKRD1 knockdown cells, this could suppress cancer metastasis. Then, we intravenously injected breast cancer cells into NOD/SCID mice (Figure 4F,G). After 28 days, the knockdown cell groups showed significantly lower liver metastasis compared to the control group (Figure 4H). Moreover, H&E results showed lower liver metastatic potential (cancer area) of LM2-ANKRD1 knockdown cells compared to control cells (Figure 4I,J), suggesting a role of ANKRD1 in breast cancer metastasis.

### 3.5. MAGE-A6 Acts Downstream of ANKRD1

To identify the downstream pathway of ANKRD1, we performed RNA-SEQ of the LM2-ANKRD1 knockdown cells as well as control cells. Differential expression analysis showed that several genes including *ipo11-lrrc70*, *eif4ebp3*, *tmem256-plscr3*, *csf3*, *il1B*, *Il8*, c*xcl2*,3 and *csf2* are upregulated and genes including *dst*, snora63, *nudp4P1*, *ankrd1*, *mageA6*, *snorA40*, *snorA8*, *serf1A* and *atp6V1G2-ddx398* are downregulated.

**Figure 5:**
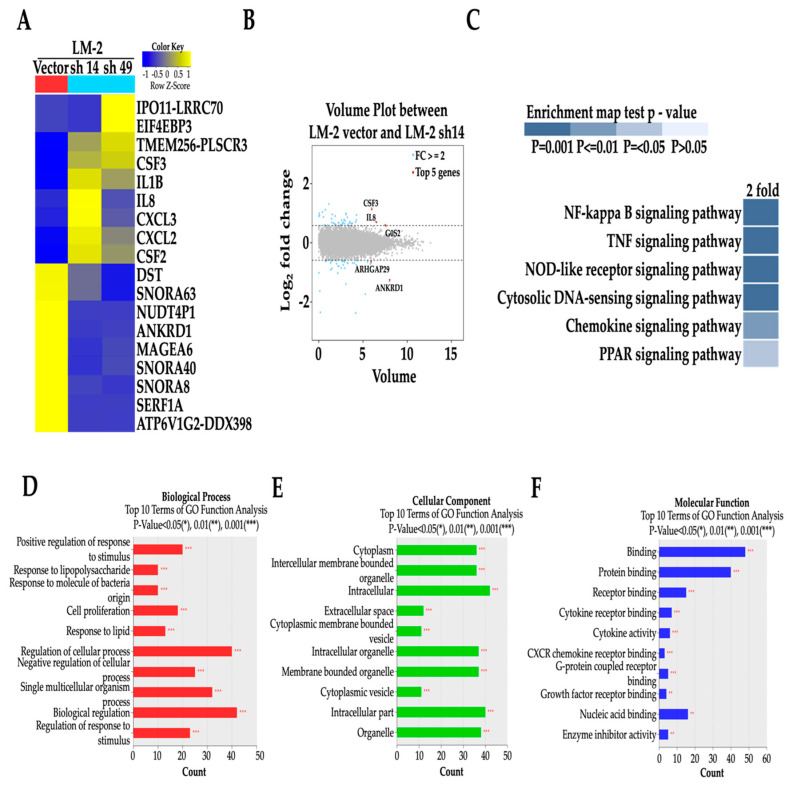
Impact of ANKRD1 knockdown on signaling pathway, biological process, cellular component and molecular function of LM-2 breast cancer cells. (**A**) Heatmap of differentially expressed genes obtained by RNA-SEQ comparing control (Vector) and LM-2-ANKRD1 knockdown cells (sh14). (**B**) Top 5 genes represented in volume plot between control and LM-2-ANKRD1 knockdown cells from RNA-SEQ. (**C**) Signaling pathways between control and LM-2-ANKRD1 knockdown cells from RNA-SEQ. (**D**) Biological processes (red) between control and LM-2-ANKRD1 knockdown cells from RNA-SEQ. (**E**) Cellular components (green) between control and LM-2-ANKRD1 knockdown cells from RNA-SEQ. (**F**) Molecular functions (blue) between control and LM-2-ANKRD1 knockdown cells from RNA-SEQ. Vector: LM-2 with empty vector, LM-2 sh14: LM-2-ANKRD1 knockdown with sh14 and LM-2 sh49: LM-2-ANKRD1 knockdown with sh49.

Out of the fifteen thousand genes from RNA-SEQ of LM2-ANKRRD1 knockdown cells, we identified one protein that was increased in MCF-7-ANKRD1-overexpressed cells and reduced in LM2-ANKRD1 knockdown cells. This protein belonged to the MAGE protein family MAGE-A6, suggesting the potential involvement of MAGE-A6 with ANKRD1 (Figure 5A). A volume plot of control and LM-2-ANKRD1 knockdown cells also demonstrated knockdown of the expression of ANKRD1 gene (Figure 5B). According to RNA-SEQ results, the major altered pathway in LM2-ANKRD1 knockdown cells is the NF-κB pathway (Figure 5C). We also listed the top 10 results from the Gene Ontology (GO) functional analysis after ANKRD1 knockdown in the cells (Figure 5D–F).

### 3.6. MAGE-A6 Knockdown Reduces Cell Migration–Invasion

**Figure 6:**
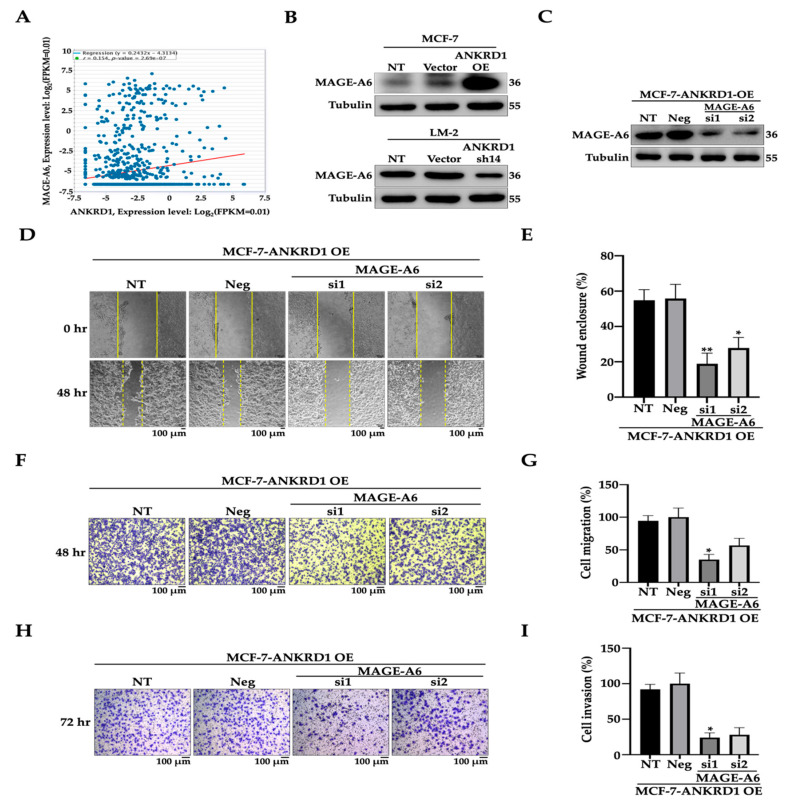
Relationship between MAGE-A6 and ANKRD1 expression and impact of MAGE-A6 silencing on migration and invasion of breast cancer cells. (**A**) Graph showing the relationship between ANKRD1 and MAGE-A6 using the TCGA database. (**B**) Immunoblots showing high expression level of MAGE-A6 protein in MCF-7-ANKRD1-overexpressed cells and low expression level in LM-2-ANKRD1 knockdown cells. (**C**) Protein expression of MAGE-A6-knockdown in MCF-7-ANKRD1-overexpressed cells. (**D**) Wound healing of MAGE-A6-knockdown cells compared to control. (**E**) Graph showing percentage of wound enclosure in MAGE-A6-knockdown cells compared to control. (**F**) Migration assay of MAGE-A6-knockdown cells compared to control. (**G**) Graph showing percentage of cell migration in MAGE-A6-knockdown cells compared to control. (**H**) Invasion assay of MAGE-A6-knockdown cells compared to control. (**I**) Graph showing percentage of cell invasion in MAGE-A6-knockdown cells compared to control. All results showed that knockdown of MAGE-A6 reduced wound healing, cell migration, and invasion. NT: parental cells without treatment; Vector: parental cells with empty vector; ANKRD1 sh14: LM-2-ANKRD1 knockdown with sh14; Neg: MCF-7-ANKRD1-overexpressing cells treated with siRNA negative control; si1: knockdown of MCF-7-ANKRD1-overexpressing cells with MAGE-A6 si1; si2: knockdown of MCF-7-ANKRD1-overexpressing cells with MAGE-A6 si2. All experiments were performed in triplicate.

To further investigate the role of MAGE-A6, we analyzed the virtual protein–protein interaction between ANKRD1 and MAGE-A6 using data from the ENCORI Pan-Cancer analysis platform (http://starbase.sysu.edu.cn) and Center for Cancer Bioinformatics (http://omics.bjcancer.org) (TCGA database). The results confirmed a positive correlation between ANKRD1 and MAGE-A6 (Figure 6A), with interaction occurring through the LRPPRC and TULP3 proteins (Figure S6A). To confirm the modulation of MAGE-A6 in ANKRD1-mediated breast cancer metastasis, we performed immunoblotting of the MAGE-A6 protein in ANKRD1-overexpressing cells. The results showed that the expression level of MAGE-A6 was increased in MCF7-ANKRD1-overexpressed cells, while it was reduced in LM2-ANKRD1 knockdown cells (Figure 6B). We also performed knockdown of MAGE-A6 in MCF-7-ANKRD1-overexpressed cells using two MAGE-A6 siRNAs with different MAGE-A6 sequences (Figure 6C) and assessed the ability of wound healing, migration and invasion. The results showed significantly reduced invasiveness of MAGE-A6 knockdown cells (Figure 6D–I). These findings indicated the involvement of MAGE-A6 in cancer migration and invasion.

### 3.7. Recombinant MAGE-A6 Induces Cell Migration–Invasion

**Figure 7:**
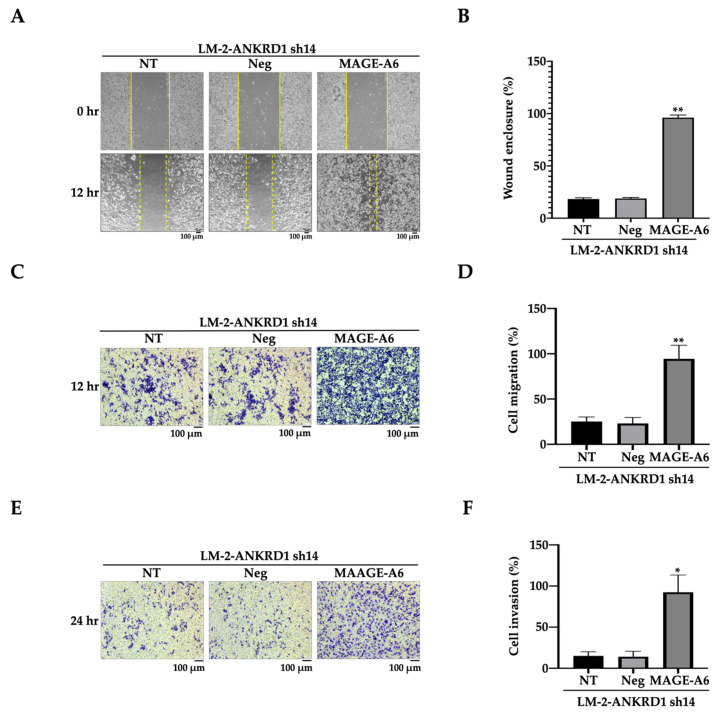
Recombinant MAGE-A6 increases migration and invasion of breast cancer cells. (**A**) Recombinant MAGE-A6 induced wound healing in LM-2-ANKRD1 knockdown cells compared to control. (**B**) Graph showing the percentage of wound closure in MAGE-A6-treated cells compared to control. All results showed knockdown of MAGE-A6. (**C**) Recombinant MAGE-A6 induced cell migration in LM-2-ANKRD1 knockdown cells compared to control. (**D**) Graph showing percentage of cell migration in MAGE-A6-treated cells compared to control. (**E**) Recombinant MAGE-A6 induced cell invasion in LM-2-ANKRD1 knockdown cells compared to control. (**F**) Graph showing percentage of cell invasion in MAGE-A6-treated cells compared to control. All the results showed that MAGE-A6 recombinant protein induced wound healing, cell migration, and invasion. NT: LM-2-ANKRD1 sh14; Neg: LM-2-ANKRD1 sh14 treated with negative control; MAGE-A6: LM-2-ANKRD1 sh14 treated with MAGE-A6 recombinant protein. All experiments were performed in triplicate.

To confirm the involvement of MAGE-A6 in cancer migration and invasion, we treated LM2-ANKRD1 knockdown cells with MAGE-A6 recombinant protein. The results showed significant increases in wound healing, cell migration and invasion in LM2-ANKRD1 knockdown cells (Figure 7A–F). The above experiments indicated that MAGE-A6 increases the potential for cell aggressiveness.

### 3.8. MAGE-A6 Acts as Downstream of NF-κB Pathway

**Figure 8:**
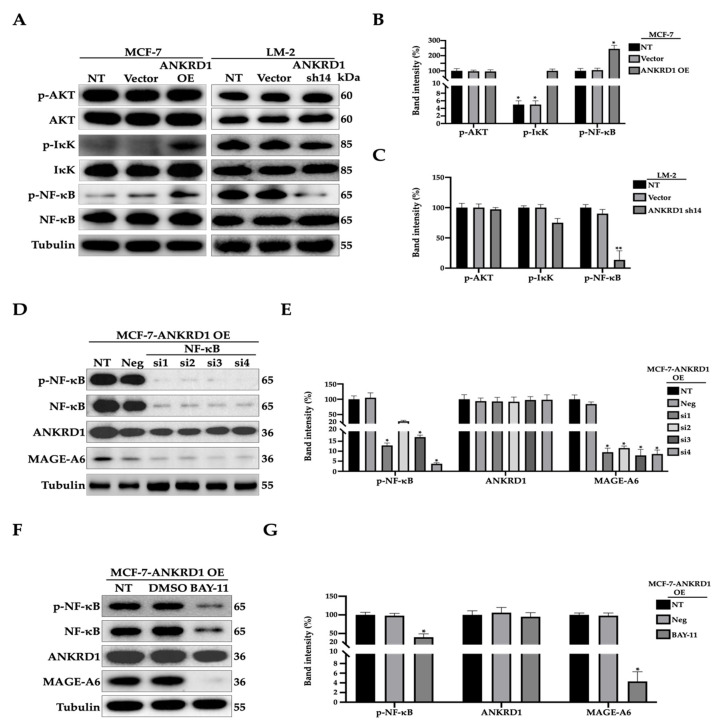
ANKRD1 regulates MAGE-A6 expression through the NF-κB pathway. (**A**–**C**) The expression level and percent band intensity of total and phosphorylated AKT/I-κK/NF-κB in MCF-7-ANKRD1-overexpressed and LM-2-ANKRD1 knockdown cells. The results show increased expression of phosphorylated IκK and NF-κB in ANKRD1 OE cells and reduced expression in ANKRD1 knockdown cells compared to NT and Vector. (**D**,**E**) Expression level and percentage of band intensity of total and phosphorylated NF-κB, ANKRD1, and MAGE-A6 in NF-κB-knockdown cells. (**F**,**G**) Expression level and percent band intensity of total and phosphorylated NF-κB, ANKRD1, and MAGE-A6 in MCF-7-ANKRD1-overexpressed cells treated with 2 µm of NF-κB inhibitor (BAY-11). The results from both siNF-κBs and NF-κB inhibitor showed no change in the expression of ANKRD1 after knockdown of NF-κB, while the expression of MAGE-A6 was reduced. NT: non-treated cells; Vector: MCF-7 or LM-2 with empty vector; ANKRD1 OE: MCF-7-ANKRD1 overexpression; sh14: LM-2-ANKRD1 knockdown with sh14; Neg: MCF-7-ANKRD1 overexpression treated with siRNA negative control; si1: MCF-7-ANKRD1 overexpression knockdown with NF-κB-siRNA1; si2: MCF-7-ANKRD1 overexpression knockdown with NF-κB-siRNA2; si3: MCF-7-ANKRD1 overexpression knockdown with NF-κB-siRNA3; si4: MCF-7-ANKRD1 overexpression knockdown with NF-κB-siRNA4; DMSO: MCF-7-ANKRD1 overexpression treated with DMSO; BAY-11: MCF-7-ANKRD1 overexpression treated with BAY-11. All experiments were performed in triplicate.

To further validate whether NF-κB is actively involved in ANKRD1 pathways, we performed Western blotting for the major NF-κB signaling molecules in ANKRD1-overexpressing cells. Even though phosphorylation of AKT, a known upstream protein of the NF-κB, was unchanged in MCF7-ANKRD1-overexpressed as well as LM2-ANKRD1 knockdown cells, the phosphorylation of IκKα and NF-κB was significantly decreased in knockdown cells and increased in overexpressed cells (Figure 8A–C). These results suggest that ANKRD1 is a regulator of IκKα and NF-κB. It has been reported that MAGE-A6 is related to the NF-κB pathway [51]; however, the underlying mechanism is not clear. To study the relationship among ANKRD1, MAGE-A6, and NF-κB, we knocked down NF-κB using NF-κB inhibitor (BAY-11) (Sigma) in MCF7-ANKRD1-overexpressed cells. The results revealed that both knockdown of NF-κB and treatment with NF-κB inhibitor reduced the expression of MAGE-A6 but not that of ANKRD1 (Figure 8D–G).

This suggested that ANKRD1 is upstream of NF-κB and that MAGE-A6 acts downstream of the NF-κB pathway.

### 3.9. MAGE-A6 Leads to Poor Prognosis in Breast Cancer

To uncover the involvement of MAGE-A6 in breast cancer prognosis, we analyzed MAGE-A6 expression in breast cancer patients (Figure S7) and found a positive correlation between high expression of MAGE-A6 and poor prognosis (low survival rate) in lymph-node-positive patients (Figure S7D), but not in lymph-node-negative patients and triple-negative breast cancer (Figure S7B,D). These results indicate that breast cancer patients who have cancer cells with high MAGE-A6 expression tend to have higher rates of lymph node metastasis compared to those with low MAGE-A6 expression. A high rate of lymph node metastasis can lead to more distant metastases that can cause mortality in cancer patients. Since these KM-plot results strongly supported our findings of the pro-metastatic role of MAGE-A6 in breast cancer cells, this could relate our in vitro findings to clinical evidence.

## 4. Discussion

In this study, we have identified ANKRD1 as a potential biomarker of poor-prognosis breast cancer metastasis and elucidated its downstream pathway in breast cancer cells. Data from microarray showed high expression of ANKRD1 in highly metastatic breast cancer cells compared to low metastatic breast cancer cells. We performed overexpression of ANKRD1 in non-metastatic breast cancer cells (MCF7 and T47D) and knockdown of ANKRD1 in highly metastatic breast cancer cells (LM2). We further studied how ANKRD1 affects cancer migration and invasion. RNA-sequencing showed that a total of 18 genes and 6 pathways were altered in ANRKD1 knockdown cells, but only 1 gene, MAGE-A6, was significantly changed at the transcriptional and translational levels. Additionally, NF-κB was increased in ANKRD1-overexpressing cells but reduced in ANKRD1 knockdown cells. This suggested that NF-κB and MAGE-A6 are downstream of ANKRD1. Furthermore, the in vivo mouse model and immunohistochemistry using a human breast cancer tissue array showed higher expression of ANKRD1 in high-grade breast cancer compared to low-grade breast cancer.

The ANKRD1 gene or CARP protein is typically upregulated in cardiac hypertrophy and heart failure. ANKRD1 is mainly expressed in cardiac muscle and is involved in many biological processes such as neovascularization and cardiomyogenesis during tissue repair. Enhanced expression of ANKRD1 is observed in animals during wound healing [52], suggesting its involvement in tissue development. ANKRD1 is one of the markers of cardiac muscle cell lineage and is involved in angiogenesis [53,54]. Moreover, ANKRD1 has been reported to be a negative transcriptional co-factor of YB-1 [36], co-factor of NF-κB, p53, and GATA-4 [30,43] and has been shown to cause changes in cardiac actin, myosin light chain and skeletal actin [55].

Karinna et al. reported that ANKRD1 associates with nucleolin at the MMP13-activator protein 1 site (AP-1). In the presence of ANKRD1, it binds to the MMP13-AP-1 site and prevents the transcription of MMP13 in fibroblast cells. The deletion of ANKRD1 may affect MMP10 transcription and the remodeling of MMPs in the ECM [52]. Breast cancer is estimated by certain characteristics of cancer, for example, grade or stage. The grade of cancer is a description of how the cancer cells look compared to normal cells. Low-grade cancer means the cancer cells look similar to normal cells. Low-grade tumors also have better prognosis because they have low invasiveness and aggressiveness. High-grade cancer means that the cancer cells look more abnormal and have greater invasiveness and aggressiveness [56]. In this study, we demonstrate that the ANKRD1 gene or CARP protein is significantly upregulated in high-grade human breast cancer tissues compared to low-grade tissues. Most of all, ANKRD1 was significantly downregulated in the breast cancer cells that had low metastatic capacity. Overexpression and knockdown of ANKRD1 were found to increase and decrease cell migration and invasion, respectively. These data are consistent with a previous study in which ANKRD1 was shown to enhance wound healing in fibroblasts [57]. Furthermore, the survival data from the Kaplan–Meier survival analysis of distant metastasis-free survival (DMFS) breast cancer patients with metastatic disease suggested that breast cancer patients with higher ANKRD1 expression have lower survival rates. These results indicate that ANKRD1 is a potential activator of metastasis of breast cancer.

In the mouse experiments, LM2-ANKRD1 knockdown cells and control vector (empty vector) cells were injected into mice either orthotopically or intravenously as previously reported [58,59]. The results showed no significant difference in primary tumor size between mice injected with LM2-ANKRD1 knockdown cells or control vector cells. However, cancer cells in the control vector exhibited greater metastasis to the liver and lungs compared to LM2-ANKRD1 knockdown cells. These results are consistent with other studies suggesting that ANKRD1 promotes cancer metastasis [60,61].

A study reported that ANKRD1 acts through the YAP pathway to promote cell mobility, tumorigenesis, and invasion in pancreatic cancer [62]. We confirmed that ANKRD1 promotes breast tumor metastasis, as the LM-2-ANKRD1 knockdown group showed lower signals in metastatic organs such as the lung and liver than the control vector group. These data suggest that the suppression of ANKRD1 can reduce metastasis. In addition, we also observed strong ANKRD1 signals in high-grade human breast tumor tissues. These results strongly suggest a significant role of ANKRD1 in tumorigenesis, cancer aggressiveness, and cancer metastasis.

To study the underlying mechanism associated with ANKRD1, we performed RNA-SEQ with LM2 cells and ANKRD1-knockdown LM2 cells and found significant downregulation of a protein called MAGE-A6. MAGE-A6 is found in most eukaryotes and belongs to the MAGE family, which can be classified into two classes depending on the tissue expression pattern [63,64]. MAGE-A6 has been reported to play a role in tumorigenesis and is linked to cancer aggressiveness, cell proliferation, cancer metastasis, and worse clinical prognoses [65,66,67,68]. Knockdown of MAGEA6 was shown to reduce tumor growth in mice [68,69]. It also appears to be involved in the mutation of p53. The MAGE-A6-TRIM28 complex has been reported to have the ability to ubiquitinate the alpha catalytic subunit of AMPK [70,71,72].

Evidence shows that MAGE-A6 increases the tumorigenicity of non-cancer cells and is also involved in the survival of cancer cells [71]. This protein is an interesting biomarker in cancer since it is expressed only in tumor cells such as lung cancer, breast cancer and multiple myeloma [65,73,74,75,76]. To study the association between ANKRD1 and MAGE-A6, we tested some of the key components in several pathways including AKT, IκKα, and NF-κB, which showed differential expression levels based on RNA-SEQ. Knockdown of ANKRD1 in highly metastatic breast tumor cells reduced the phosphorylation of IκKα and NF-κB. As expected, the phosphorylation of these markers was increased in MCF7-ANKRD1-overexpressing non-metastatic breast tumor cells. This is consistent with several previous studies showing that NF-κB targets many proteins and acts as a transcription factor, resulting in the promotion of cancer metastasis [77,78,79,80].

We next studied the role of MAGE-A6 in cancer metastasis. Interestingly, the RNA and protein expression levels of MAGE-A6 in MCF7-ANKRD1-overexpressed and LM2-ANKRD1 knockdown cells were increased and decreased, respectively. Wound healing, migration and invasion were generally used to test the effect of cancer metastasis. Knockdown of MAGE-A6 in MCF7-ANKRD1-overexpressed cells reduced cell migration and invasion. On the other hand, LM2-ANKRD1 knockdown cells treated with MAGE-A6 recombinant protein showed increased cell migration and invasion. These results are supported by previous studies in which the overexpression of MAGE-A6 was found to increase cell migration; in addition, MAGE-A6 knockdown was shown to reduce migration of glioma and esophageal cancer cells [81,82]. To study the association between ANKRD1, NF-κB and MAGE-A6, we performed knockdown of NF-κB and also treated MCF-7-ANKRD1-overexpressed cells with NF-κB inhibitor to understand the pathway. Moreover, inhibiting the expression of NF-κB reduced the expression level of MAGE-A6, while the expression of ANKRD1 remained unchanged in both experiments. This indicated that NF-κB may be a downstream signaling molecule of ANKRD1 and upstream signaling molecule of MAGE-A6. This is consistent with several studies showing that NF-κB targets MAGE-A6 [83,84,85].

This result indicates that ANKRD1 affects MAGE-A6 through the NF-κB pathway. NF-κB is a transcription factor involved in a variety of biological processes. A growing body of evidence supports the role of NF-κB in oncogenesis, especially cancer cell proliferation, migration, and invasion. Elucidating the functional aspects of NF-κB activation and the underlying mechanisms can facilitate the development of novel targeted therapies for cancer [86,87]. In addition, Pires et al. reported that NF-κB directly promotes metastasis of breast cancer through the transcriptional activation of EMT regulator genes [88]. Moreover, several recent studies have suggested that the inhibition of MAGE-A6 reduces metastasis [89,90,91,92]. It is also known that the contribution of MAGE-A6 to tumorigenesis is via its direct interaction with p53 resulting in the loss–gain of cancer metastasis [93,94]. MAGE-A6 has also been reported to be phosphorylated by IκKα kinase and to interact with ubiquitin ligase for its degradation, especially in breast cancer [49]. This leads to altered cell proliferation, apoptosis, metastasis, and tumorigenesis [95,96,97,98].

**Figure 9:**
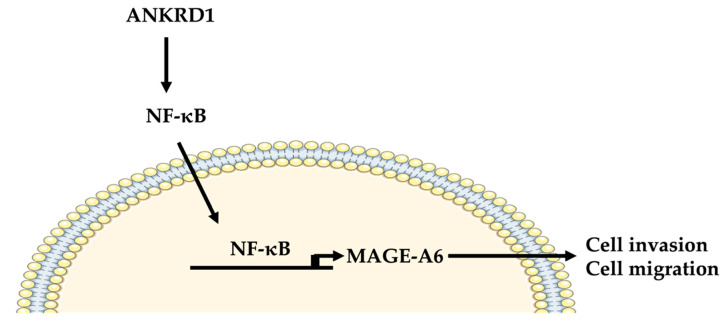
A proposed pro-metastatic mechanism of ANKRD1 in breast cancer cells. ANKRD1 promotes activation of NF-κB, which upregulates MAGE-A6 expression. Increased expression of MAGE-A6 causes an increase in cell migration and invasion. Parts of the figure are adapted from Servier Medical Art, licensed by Servier under a Creative Commons Attribution 3.0 Unported License (https://creativecommons.org/licenses/by/3.0/).

Therefore, we proposed that ANKRD1 is an upstream regulator of NF-κB-MAGE-A6, and results suggest that ANKRK1 could be a potential therapeutic target. Our studies showed that ANKRD1 allows the activation of IκKα–NF-κB-MAGE-A6, leading to breast cancer metastasis (see scheme in Figure 9). The relevance of the ANKRD1-NF-κB-MAGE-A6 axis in breast cancer is its pro-metastatic role. In addition, high expression of MAGE-A6 showed increased breast cancer migration and invasion, which is consistent with the KM-plot results showing that lymph-node-positive breast cancer has a low survival rate. This is a leading cause of death in breast cancer patients. Therefore, ANKRD1 and MAGE-A6 may be used as targets for anti-metastatic therapy of breast cancer in the future.

## 5. Conclusions

In conclusion, we found that ANKRD1 is associated with breast cancer metastasis. It is highly expressed in highly metastatic breast cancer cell lines compared to the non-metastatic breast cancer cell lines as well as the normal breast cells. Our findings suggest that ANKRD1 may play a significant role in breast cancer migration and metastasis by regulating the NF-κB-MAGE-A6 cascade.

## Figures and Tables

**Table 1:** Semi-quantitative analysis of immunohistochemical staining of ANKRD1 in different tumor grades of breast cancer tissues.

	Grade 1 (Number of Samples = 7)		Grade 2 (Number of Samples = 58)		Grade 3 (Number of Samples = 35)		*p*-Value
	Pathologists	ImageJ	Pathologists	ImageJ	Pathologists	ImageJ	
**Staining** **intensity**	+	0.19 ± 0.001	++	0.65 ± 0.185	+++	16.93 ± 2.523	<0.001

## Data Availability

All data generated or analyzed during this study are included in this published article and its additional files. The RNA-seq used in this study is available in the GEO database under accession number GSE273077.

## Supplementary Materials

The following supporting information can be downloaded at https://www.mdpi.com/article/10.3390/cancers16193306/s1: Table S1: Primer list for real-time PCR; Table S2: List of siRNAs used in the study; Figure S1: “ANKRD1” gene in TCGA on the database in breast cancer using Kaplan–Meier survival analysis (mRNA gene chip); Figure S2: Comparison of migration and adhesion and cancer associated fibroblasts markers between NT (MCF-7), Vector and MCF-7-ANKRD1-overexpressed cells by real-time PCR; Figure S3: Overexpression of ANKRD1 induces cell migration and invasion in T47D, a non-metastatic breast cancer cell line; Figure S4: Comparison of migration and adhesion and cancer-associated fibroblast markers between NT (LM-2), Vector and LM-2-ANKRD1-knockdown cells by real-time PCR; Figure S5: Comparison of the radiant efficacy of different organs at day 28 in vivo metastasis models; Figure S6: Protein–protein interaction between ANKRD1 and MAGE-A6; Figure S7: “MAGE-A6” gene in TCGA on the database in breast cancer using Kaplan–Meier survival analysis (mRNA gene chip).

## References

[1] Lei S., Zheng R., Zhang S., Wang S., Chen R., Sun K., Zeng H., Zhou J., Wei W. (2021). Global Patterns of Breast Cancer Incidence and Mortality: A Population-Based Cancer Registry Data Analysis from 2000 to 2020. Cancer Commun..

[2] Sung H., Ferlay J., Siegel R.L., Laversanne M., Soerjomataram I., Jemal A., Bray F. (2021). Global Cancer Statistics 2020: GLOBOCAN Estimates of Incidence and Mortality Worldwide for 36 Cancers in 185 Countries. CA Cancer J. Clin..

[3] Goncalves H., Guerra M.R., Duarte Cintra J.R., Fayer V.A., Brum I.V., Bustamante Teixeira M.T. (2018). Survival Study of Triple-Negative and Non-Triple-Negative Breast Cancer in a Brazilian Cohort. Clin. Med. Insights Oncol..

[4] Lebert J.M., Lester R., Powell E., Seal M., McCarthy J. (2018). Advances in the Systemic Treatment of Triple-Negative Breast Cancer. Curr. Oncol..

[5] Anders C., Carey L.A. (2008). Understanding and Treating Triple-Negative Breast Cancer. Oncology.

[6] Anders C.K., Abramson V., Tan T., Dent R. (2016). The Evolution of Triple-Negative Breast Cancer: From Biology to Novel Therapeutics. Am. Soc. Clin. Oncol. Educ. Book.

[7] Diana A., Franzese E., Centonze S., Carlino F., della Corte C.M., Ventriglia J., Petrillo A., de Vita F., Alfano R., Ciardiello F. (2018). Triple-Negative Breast Cancers: Systematic Review of the Literature on Molecular and Clinical Features with a Focus on Treatment with Innovative Drugs. Curr. Oncol. Rep..

[8] Plasilova M.L., Hayse B., Killelea B.K., Horowitz N.R., Chagpar A.B., Lannin D.R. (2016). Features of Triple-Negative Breast Cancer: Analysis of 38,813 Cases from the National Cancer Database. Medicine.

[9] Yao H., He G., Yan S., Chen C., Song L., Rosol T.J., Deng X. (2017). Triple-Negative Breast Cancer: Is There a Treatment on the Horizon?. Oncotarget.

[10] O’Toole S.A., Beith J.M., Millar E.K., West R., McLean A., Cazet A., Swarbrick A., Oakes S.R. (2013). Therapeutic Targets in Triple Negative Breast Cancer. J. Clin. Pathol..

[11] Perou C.M. (2011). Molecular Stratification of Triple-Negative Breast Cancers. Oncologist.

[12] Mouh F.Z., Mzibri M.E., Slaoui M., Amrani M. (2016). Recent Progress in Triple Negative Breast Cancer Research. Asian Pac. J. Cancer Prev..

[13] Klein C.A. (2009). Parallel Progression of Primary Tumours and Metastases. Nat. Rev. Cancer.

[14] Qiu J., Xue X., Hu C., Xu H., Kou D., Li R., Li M. (2016). Comparison of Clinicopathological Features and Prognosis in Triple-Negative and Non-Triple Negative Breast Cancer. J. Cancer.

[15] Davion S.M., Siziopikou K.P., Sullivan M.E. (2012). Cytokeratin 7: A Re-Evaluation of the “tried and True” in Triple-Negative Breast Cancers. Histopathology.

[16] Valastyan S., Weinberg R.A. (2011). Tumor Metastasis: Molecular Insights and Evolving Paradigms. Cell.

[17] Talmadge J.E., Fidler I.J. (2010). AACR Centennial Series: The Biology of Cancer Metastasis: Historical Perspective. Cancer Res..

[18] Hanahan D., Weinberg R.A. (2011). Hallmarks of Cancer: The next Generation. Cell.

[19] Fares J., Fares M.Y., Khachfe H.H., Salhab H.A., Fares Y. (2020). Molecular Principles of Metastasis: A Hallmark of Cancer Revisited. Signal Transduct. Target. Ther..

[20] De Craene B., Berx G. (2013). Regulatory Networks Defining EMT during Cancer Initiation and Progression. Nat. Rev. Cancer.

[21] Kikuchi M., Yamashita K., Waraya M., Minatani N., Ushiku H., Kojo K., Ema A., Kosaka Y., Katoh H., Sengoku N. (2016). Epigenetic Regulation of ZEB1-RAB25/ESRP1 Axis Plays a Critical Role in Phenylbutyrate Treatment-Resistant Breast Cancer. Oncotarget.

[22] Takahashi A., Seike M., Chiba M., Takahashi S., Nakamichi S., Matsumoto M., Takeuchi S., Minegishi Y., Noro R., Kunugi S. (2018). Ankyrin Repeat Domain 1 Overexpression Is Associated with Common Resistance to Afatinib and Osimertinib in EGFR-Mutant Lung Cancer. Sci. Rep..

[23] Miller M.K., Bang M.-L., Witt C.C., Labeit D., Trombitas C., Watanabe K., Granzier H., McElhinny A.S., Gregorio C.C., Labeit S. (2003). The Muscle Ankyrin Repeat Proteins: CARP, Ankrd2/Arpp and DARP as a Family of Titin Filament-Based Stress Response Molecules. J. Mol. Biol..

[24] Bang M.L., Mudry R.E., McElhinny A.S., Trombitás K., Geach A.J., Yamasaki R., Sorimachi H., Granzier H., Gregorio C.C., Labeit S. (2001). Myopalladin, a Novel 145-Kilodalton Sarcomeric Protein with Multiple Roles in Z-Disc and I-Band Protein Assemblies. J. Cell Biol..

[25] Gnimassou O., Francaux M., Deldicque L. (2017). Hippo Pathway and Skeletal Muscle Mass Regulation in Mammals: A Controversial Relationship. Front. Physiol..

[26] Maugeri-Sacca M., de Maria R. (2018). The Hippo Pathway in Normal Development and Cancer. Pharmacol. Ther..

[27] Liu Y., Liu H., Meyer C., Li J., Nadalin S., Königsrainer A., Weng H., Dooley S., Ten Dijke P. (2013). Transforming Growth Factor-β (TGF-β)-Mediated Connective Tissue Growth Factor (CTGF) Expression in Hepatic Stellate Cells Requires Stat3 Signaling Activation. J. Biol. Chem..

[28] Gressner O.A., Lahme B., Demirci I., Gressner A.M., Weiskirchen R. (2007). Differential Effects of TGF-Beta on Connective Tissue Growth Factor (CTGF/CCN2) Expression in Hepatic Stellate Cells and Hepatocytes. J. Hepatol..

[29] Zou Y., Evans S., Chen J., Kuo H.C., Harvey R.P., Chien K.R. (1997). CARP, a Cardiac Ankyrin Repeat Protein, Is Downstream in the Nkx2-5 Homeobox Gene Pathway. Development.

[30] Kojic S., Nestorovic A., Rakicevic L., Belgrano A., Stankovic M., Divac A., Faulkner G. (2010). A Novel Role for Cardiac Ankyrin Repeat Protein Ankrd1/CARP as a Co-Activator of the P53 Tumor Suppressor Protein. Arch. Biochem. Biophys..

[31] Zhang N., Ye F., Zhu W., Hu D., Xiao C., Nan J., Su S., Wang Y., Liu M., Gao K. (2016). Cardiac Ankyrin Repeat Protein Attenuates Cardiomyocyte Apoptosis by Upregulation of Bcl-2 Expression. Biochim. Biophys. Acta.

[32] Ling S.S.M., Chen Y.-T., Wang J., Richards A.M., Liew O.W. (2017). Ankyrin Repeat Domain 1 Protein: A Functionally Pleiotropic Protein with Cardiac Biomarker Potential. Int. J. Mol. Sci..

[33] Liu X.-H., Bauman W.A., Cardozo C. (2015). ANKRD1 Modulates Inflammatory Responses in C2C12 Myoblasts through Feedback Inhibition of NF-ΚB Signaling Activity. Biochem. Biophys. Res. Commun..

[34] Zhong L., Chiusa M., Cadar A.G., Lin A., Samaras S., Davidson J.M., Lim C.C. (2015). Targeted Inhibition of ANKRD1 Disrupts Sarcomeric ERK-GATA4 Signal Transduction and Abrogates Phenylephrine-Induced Cardiomyocyte Hypertrophy. Cardiovasc. Res..

[35] Torrado M., Nespereira B., López E., Centeno A., Castro-Beiras A., Mikhailov A.T. (2005). ANKRD1 Specifically Binds CASQ2 in Heart Extracts and Both Proteins Are Co-Enriched in Piglet Cardiac Purkinje Cells. J. Mol. Cell. Cardiol..

[36] Franklin J.M., Ghosh R.P., Shi Q., Reddick M.P., Liphardt J.T. (2020). Concerted Localization-Resets Precede YAP-Dependent Transcription. Nat. Commun..

[37] Jiménez A.P., Traum A., Boettger T., Hackstein H., Richter A.M., Dammann R.H. (2017). The Tumor Suppressor RASSF1A Induces the YAP1 Target Gene ANKRD1 That Is Epigenetically Inactivated in Human Cancers and Inhibits Tumor Growth. Oncotarget.

[38] Moon S., Kim W., Kim S., Kim Y., Song Y., Bilousov O., Kim J., Lee T., Cha B., Kim M. (2017). Phosphorylation by NLK Inhibits YAP-14-3-3-Interactions and Induces Its Nuclear Localization. EMBO Rep..

[39] Lange S., Gehmlich K., Lun A.S., Blondelle J., Hooper C., Dalton N.D., Alvarez E.A., Zhang X., Bang M.-L., Abassi Y.A. (2016). MLP and CARP Are Linked to Chronic PKCα Signalling in Dilated Cardiomyopathy. Nat. Commun..

[40] Murphy N.P., Lubbers E.R., Mohler P.J. (2020). Advancing Our Understanding of AnkRD1 in Cardiac Development and Disease. Cardiovasc. Res..

[41] Francisco J., Zhang Y., Jeong J.I., Mizushima W., Ikeda S., Ivessa A., Oka S., Zhai P., Tallquist M.D., Del Re D.P. (2020). Blockade of Fibroblast YAP Attenuates Cardiac Fibrosis and Dysfunction Through MRTF-A Inhibition. JACC Basic Transl. Sci..

[42] Song Y., Xu J., Li Y., Jia C., Ma X., Zhang L., Xie X., Zhang Y., Gao X., Zhang Y. (2012). Cardiac Ankyrin Repeat Protein Attenuates Cardiac Hypertrophy by Inhibition of ERK1/2 and TGF-β Signaling Pathways. PLoS ONE.

[43] Xu Z., Lu D., Yuan J., Wang L., Wang J., Lei Z., Liu S., Wu J., Wang J., Huang L. (2022). Storax Attenuates Cardiac Fibrosis Following Acute Myocardial Infarction in Rats via Suppression of AT1R-Ankrd1-P53 Signaling Pathway. Int. J. Mol. Sci..

[44] Cui M., Wang Z., Chen K., Shah A.M., Tan W., Duan L., Sanchez-Ortiz E., Li H., Xu L., Liu N. (2020). Dynamic Transcriptional Responses to Injury of Regenerative and Non-Regenerative Cardiomyocytes Revealed by Single-Nucleus RNA Sequencing. Dev. Cell.

[45] Lee M.J., Kwak Y.K., You K.R., Lee B.H., Kim D.G. (2009). Involvement of GADD153 and Cardiac Ankyrin Repeat Protein in Cardiac Ischemia-Reperfusion Injury. Exp. Mol. Med..

[46] Bogomolovas J., Brohm K., Čelutkienė J., Balčiūnaitė G., Bironaitė D., Bukelskienė V., Daunoravičus D., Witt C.C., Fielitz J., Grabauskienė V. (2015). Induction of Ankrd1 in Dilated Cardiomyopathy Correlates with the Heart Failure Progression. BioMed Res. Int..

[47] Jensen N.F., Stenvang J., Beck M.K., Hanakova B., Belling K.C., Do K.N., Viuff B., Nygard S.B., Gupta R., Rasmussen M.H. (2015). Establishment and Characterization of Models of Chemotherapy Resistance in Colorectal Cancer: Towards a Predictive Signature of Chemoresistance. Mol. Oncol..

[48] Lei Y., Henderson B.R., Emmanuel C., Harnett P.R., deFazio A. (2015). Inhibition of ANKRD1 Sensitizes Human Ovarian Cancer Cells to Endoplasmic Reticulum Stress-Induced Apoptosis. Oncogene.

[49] Scurr L.L., Guminski A.D., Chiew Y.E., Balleine R.L., Sharma R., Lei Y., Pryor K., Wain G.V., Brand A., Byth K. (2008). Ankyrin Repeat Domain 1, ANKRD1, a Novel Determinant of Cisplatin Sensitivity Expressed in Ovarian Cancer. Clin. Cancer Res..

[50] Rakha E.A., Tse G.M., Quinn C.M. (2023). An Update on the Pathological Classification of Breast Cancer. Histopathology.

[51] Florke Gee R.R., Chen H., Lee A.K., Daly C.A., Wilander B.A., Fon Tacer K., Potts P.R. (2020). Emerging Roles of the MAGE Protein Family in Stress Response Pathways. J. Biol. Chem..

[52] Almodovar-Garcia K., Kwon M., Samaras S.E., Davidson J.M. (2014). ANKRD1 Acts as a Transcriptional Repressor of MMP13 via the AP-1 Site. Mol. Cell. Biol..

[53] Kuo H., Chen J., Ruiz-Lozano P., Zou Y., Nemer M., Chien K.R. (1999). Control of Segmental Expression of the Cardiac-Restricted Ankyrin Repeat Protein Gene by Distinct Regulatory Pathways in Murine Cardiogenesis. Development.

[54] Shi Y., Reitmaier B., Regenbogen J., Slowey R.M., Opalenik S.R., Wolf E., Goppelt A., Davidson J.M. (2005). CARP, a Cardiac Ankyrin Repeat Protein, Is up-Regulated during Wound Healing and Induces Angiogenesis in Experimental Granulation Tissue. Am. J. Pathol..

[55] Jeyaseelan R., Poizat C., Baker R.K., Abdishoo S., Isterabadi L.B., Lyons G.E., Kedes L. (1997). A Novel Cardiac-Restricted Target for Doxorubicin. CARP, a Nuclear Modulator of Gene Expression in Cardiac Progenitor Cells and Cardiomyocytes. J. Biol. Chem..

[56] Henson D.E., Ries L., Freedman L.S., Carriaga M. (1991). Relationship among Outcome, Stage of Disease, and Histologic Grade for 22,616 Cases of Breast Cancer. The Basis for a Prognostic Index. Cancer.

[57] Samaras S.E., Almodovar-Garcia K., Wu N., Yu F., Davidson J.M. (2015). Global Deletion of Ankrd1 Results in a Wound-Healing Phenotype Associated with Dermal Fibroblast Dysfunction. Am. J. Pathol..

[58] Elkin M., Vlodavsky I. (2001). Tail Vein Assay of Cancer Metastasis. Curr. Protoc. Cell Biol..

[59] Werbeck J.L., Thudi N.K., Martin C.K., Premanandan C., Yu L., Ostrowksi M.C., Rosol T.J. (2014). Tumor Microenvironment Regulates Metastasis and Metastasis Genes of Mouse MMTV-PymT Mammary Cancer Cells in Vivo. Vet. Pathol..

[60] Cheng Y., Hou T., Ping J., Chen T., Yin B. (2018). LMO3 Promotes Hepatocellular Carcinoma Invasion, Metastasis and Anoikis Inhibition by Directly Interacting with LATS1 and Suppressing Hippo Signaling. J. Exp. Clin. Cancer Res..

[61] Janse van Rensburg H.J., Yang X. (2016). The Roles of the Hippo Pathway in Cancer Metastasis. Cell. Signal..

[62] Yang S., Zhang L., Purohit V., Shukla S.K., Chen X., Yu F., Fu K., Chen Y., Solheim J., Singh P.K. (2015). Active YAP Promotes Pancreatic Cancer Cell Motility, Invasion and Tumorigenesis in a Mitotic Phosphorylation-Dependent Manner through LPAR3. Oncotarget.

[63] Barker P.A., Salehi A. (2002). The MAGE Proteins: Emerging Roles in Cell Cycle Progression, Apoptosis, and Neurogenetic Disease. J. Neurosci. Res..

[64] Simpson A.J.G., Caballero O.L., Jungbluth A., Chen Y.-T., Old L.J. (2005). Cancer/Testis Antigens, Gametogenesis and Cancer. Nat. Rev. Cancer.

[65] Nardiello T., Jungbluth A.A., Mei A., Diliberto M., Huang X., Dabrowski A., Andrade V.C.C., Wasserstrum R., Ely S., Niesvizky R. (2011). MAGE-A Inhibits Apoptosis in Proliferating Myeloma Cells through Repression of Bax and Maintenance of Survivin. Clin. Cancer. Res..

[66] Liu W., Cheng S., Asa S.L., Ezzat S. (2008). The Melanoma-Associated Antigen A3 Mediates Fibronectin-Controlled Cancer Progression and Metastasis. Cancer Res..

[67] Yang F., Zhou X., Miao X., Zhang T., Hang X., Tie R., Liu N., Tian F., Wang F., Yuan J. (2014). MAGEC2, an Epithelial-Mesenchymal Transition Inducer, Is Associated with Breast Cancer Metastasis. Breast Cancer Res. Treat..

[68] Brasseur F., Rimoldi D., Liénard D., Lethé B., Carrel S., Arienti F., Suter L., Vanwijck R., Bourlond A., Humblet Y. (1995). Expression of MAGE Genes in Primary and Metastatic Cutaneous Melanoma. Int. J. Cancer.

[69] Ye X., Xie J., Huang H., Deng Z. (2018). Knockdown of MAGEA6 Activates AMP-Activated Protein Kinase (AMPK) Signaling to Inhibit Human Renal Cell Carcinoma Cells. Cell. Physiol. Biochem..

[70] Pineda C.T., Ramanathan S., Fon Tacer K., Weon J.L., Potts M.B., Ou Y.H., White M.A., Potts P.R. (2015). Degradation of AMPK by a Cancer-Specific Ubiquitin Ligase. Cell.

[71] Pineda C.T., Potts P.R. (2015). Oncogenic MAGEA-TRIM28 Ubiquitin Ligase Downregulates Autophagy by Ubiquitinating and Degrading AMPK in Cancer. Autophagy.

[72] van Tongelen A., Loriot A., de Smet C. (2017). Oncogenic Roles of DNA Hypomethylation through the Activation of Cancer-Germline Genes. Cancer Lett..

[73] Tajima K., Obata Y., Tamaki H., Yoshida M., Chen Y.-T., Scanlan M.J., Old L.J., Kuwano H., Takahashi T., Takahashi T. (2003). Expression of Cancer/Testis (CT) Antigens in Lung Cancer. Lung Cancer.

[74] Ayyoub M., Scarlata C.-M., Hamaï A., Pignon P., Valmori D. (2014). Expression of MAGE-A3/6 in Primary Breast Cancer Is Associated with Hormone Receptor Negative Status, High Histologic Grade, and Poor Survival. J. Immunother..

[75] Otte M., Zafrakas M., Riethdorf L., Pichlmeier U., Löning T., Jänicke F., Pantel K. (2001). MAGE-A Gene Expression Pattern in Primary Breast Cancer. Cancer Res..

[76] Andrade V.C.C., Vettore A.L., Felix R.S., Almeida M.S.S., Carvalho F., Oliveira J.S.R., Chauffaille M.L.L.F., Andriolo A., Caballero O.L., Zago M.A. (2008). Prognostic Impact of Cancer/Testis Antigen Expression in Advanced Stage Multiple Myeloma Patients. Cancer Immun..

[77] Zhao L., Jiang L., Zhang M., Zhang Q., Guan Q., Li Y., He M., Zhang J., Wei M. (2021). NF-ΚB-Activated SPRY4-IT1 Promotes Cancer Cell Metastasis by Downregulating TCEB1 MRNA via Staufen1-Mediated MRNA Decay. Oncogene.

[78] Zhang T., Ma C., Zhang Z., Zhang H., Hu H. (2021). NF-ΚB Signaling in Inflammation and Cancer. MedComm.

[79] Wang W., Nag S.A., Zhang R. (2015). Targeting the NFκB Signaling Pathways for Breast Cancer Prevention and Therapy. Curr. Med. Chem..

[80] Harrington B.S., Annunziata C.M. (2019). NF-ΚB Signaling in Ovarian Cancer. Cancers.

[81] Pan S.-J., Ren J., Jiang H., Liu W., Hu L.-Y., Pan Y.-X., Sun B., Sun Q.-F., Bian L.-G. (2018). MAGEA6 Promotes Human Glioma Cell Survival via Targeting AMPKα1. Cancer Lett..

[82] Liu M., Li J., Wang Y., Ghaffar M., Yang Y., Wang M., Li C. (2022). MAGEA6 Positively Regulates MSMO1 and Promotes the Migration and Invasion of Oesophageal Cancer Cells. Exp. Ther. Med..

[83] Kendall S.E., Battelli C., Irwin S., Mitchell J.G., Glackin C.A., Verdi J.M. (2005). NRAGE Mediates P38 Activation and Neural Progenitor Apoptosis via the Bone Morphogenetic Protein Signaling Cascade. Mol. Cell. Biol..

[84] Matluk N., Rochira J.A., Karaczyn A., Adams T., Verdi J.M. (2010). A Role for NRAGE in NF-KappaB Activation through the Non-Canonical BMP Pathway. BMC Biol..

[85] Rochira J.A., Matluk N.N., Adams T.L., Karaczyn A.A., Oxburgh L., Hess S.T., Verdi J.M. (2011). A Small Peptide Modeled after the NRAGE Repeat Domain Inhibits XIAP-TAB1-TAK1 Signaling for NF-ΚB Activation and Apoptosis in P19 Cells. PLoS ONE.

[86] Dolcet X., Llobet D., Pallares J., Matias-Guiu X. (2005). NF-KB in Development and Progression of Human Cancer. Virchows Arch..

[87] Gaptulbarova K.A., Tsyganov M.M., Pevzner A.M., Ibragimova M.K., Litviakov N. (2020). V NF-KB as a Potential Prognostic Marker and a Candidate for Targeted Therapy of Cancer. Exp. Oncol..

[88] Pires B.R.B., Mencalha A.L., Ferreira G.M., de Souza W.F., Morgado-Díaz J.A., Maia A.M., Corrêa S., Abdelhay E.S.F.W. (2017). NF-KappaB Is Involved in the Regulation of EMT Genes in Breast Cancer Cells. PLoS ONE.

[89] Qian Z., Zhang G., Song G., Shi J., Gong L., Mou Y., Han Y. (2017). Integrated Analysis of Genes Associated with Poor Prognosis of Patients with Colorectal Cancer Liver Metastasis. Oncotarget.

[90] Laban S., Giebel G., Klümper N., Schröck A., Doescher J., Spagnoli G., Thierauf J., Theodoraki M.-N., Remark R., Gnjatic S. (2017). MAGE Expression in Head and Neck Squamous Cell Carcinoma Primary Tumors, Lymph Node Metastases and Respective Recurrences-Implications for Immunotherapy. Oncotarget.

[91] Alsalloum A., Shevchenko J.A., Sennikov S. (2023). The Melanoma-Associated Antigen Family A (MAGE-A): A Promising Target for Cancer Immunotherapy?. Cancers.

[92] Gu L., Sang M., Yin D., Liu F., Wu Y., Liu S., Huang W., Shan B. (2018). MAGE-A Gene Expression in Peripheral Blood Serves as a Poor Prognostic Marker for Patients with Lung Cancer. Thorac. Cancer.

[93] Doyle J.M., Gao J., Wang J., Yang M., Potts P.R. (2010). MAGE-RING Protein Complexes Comprise a Family of E3 Ubiquitin Ligases. Mol. Cell.

[94] Powell E., Piwnica-Worms D., Piwnica-Worms H. (2014). Contribution of P53 to Metastasis. Cancer Discov..

[95] Kwon S., Kang S.H., Ro J., Jeon C.-H., Park J.-W., Lee E.S. (2005). The Melanoma Antigen Gene as a Surveillance Marker for the Detection of Circulating Tumor Cells in Patients with Breast Carcinoma. Cancer.

[96] Brisam M., Rauthe S., Hartmann S., Linz C., Brands R.C., Kübler A.C., Rosenwald A., Müller-Richter U.D. (2016). Expression of MAGE-A1-A12 Subgroups in the Invasive Tumor Front and Tumor Center in Oral Squamous Cell Carcinoma. Oncol. Rep..

[97] Mei A.H.-C., Tung K., Han J., Perumal D., Laganà A., Keats J., Auclair D., Chari A., Jagannath S., Parekh S. (2020). MAGE-A Inhibit Apoptosis and Promote Proliferation in Multiple Myeloma through Regulation of BIM and P21Cip1. Oncotarget.

[98] Deng L., Meng T., Chen L., Wei W., Wang P. (2020). The Role of Ubiquitination in Tumorigenesis and Targeted Drug Discovery. Signal Transduct. Target. Ther..

