# Supplementary material for: ANKRD1 Promotes Breast Cancer Metastasis by Activating NF-κB-MAGE-A6 Pathway
Source: Cancers (Basel). 2024 Sep 27;16(19):3306. doi: 10.3390/cancers16193306 (PMC11476229; doi:10.3390/cancers16193306)
Supplement: Supplementary file 1 [file cancers-16-03306-s001.zip › cancers-3083127-supplementary.pdf]

Supplementary Materials

Table S1. Primer list for real time PCR.

**Gene name**		**Sequence (5′-3′)**
Gapdh	F	CTTCAACAGCGACACCCACTCCTC
	R	GTCCACCACCCTGTTGCTGTAG
ANKRD1	F	CGGAGCATCTTATCGCCTGT
	R	TTGAGATCCGCGCCATACAT
ANKRD1_cloneing	F	GACGTAAGATCTATGATGGTACTGAAAGTAGAGGAAC
	R	GGATCTGCGGCCGCTCAGAATGTAGCTATGCGAGAG
E-cadherin	F	GAACGCATTGCCACATACAC
	R	ATTCGGGCTTGTTGTCATTC
N-cadherin	F	AGGATCAACCCCATACACCA
	R	TGGTTTGACCACGGTGACTA
Vimentin	F	GAGAACTTTGCCGTTGAAGC
	R	CTCAATGTCAAGGGCCATCT
Snail1	F	TGCCCTCAAGATGCACATCCGA
	R	GGGACAGGAGAAGGGCTTCTC
Twist	F	GTCCGCAGTCTTACGAGGAG
	R	TGAATCTTGCTCAGCTTGTCC
Fibronectin	F	AAAATGGCCAGATGATGAGC
	R	TGGCACCGAGATATTCCTTC
CDC42	F	GCGATGGTGCTGTTGGTAAA
	R	TGTGGATAACTCAGCGGTCG
MYLK	F	TGCCTCGTCACACATTTCCA
	R	TCCGAGGGGGCAAAATGAAA
ROCK1	F	TGAAGGTGATTGGTAGAGGTGC
	R	GGCAAAAGCCATGATGTCCC
RAC	F	GGGAGACGGAGCTGTAGGTA
	R	CGGATAGGATAGGGGGCGTA
RHOA	F	TCGTTAGTCCACGGTCTGGT
	R	CAGCCATTGCTCAGGCAAC
CTNNB1	F	GCTGGGACCTTGCATAACCT
	R	CGCACTGCCATTTTAGCTCC
SOX2	F	GGGAAATGGGAGGGGTGCAAAAGAGG
	R	TTGCGTGAGTGTGGATGGGATTGGTG
Nanog	F	ACC AGT CCC AAA GGC AAA CA
	R	TCT GCT GGA GGC TGA GGT AT
OCT4	F	CTT CCC TCC AAC CAG TTG CCC CAA AC
	R	GGG AAA TGG GAG GGG TGC AAA AGA GG
KLF4	F	GGACACACGGGATGATGCTC
	R	TTGTGTAGGTTTTGCCGCAG
CXCL1	F	TCACAGTGTGTGGTCAACAT
	R	AGCCCCTTTGTTCTAAGCCA
CSF3	F	AGAGCTTCCTGCTCAAGTGC
	R	AGCTTGTAGGTGGCACACTC

**Gene name**		**Sequence (5′-3′)**
EIF4EBP3	F	GTCCACTAGCTGCCCGATTC
	R	CTCCAGCAGGAACTTTCGGT
IL1B	F	GTACCTGTCCTGCGTGTTGA
	R	GGGAACTGGGCAGACTCAAA
IPO11	F	CAGGCCACCAGTCAGGATAC
	R	GCTACACGTCTCCAGTAGCG
LRRC70	F	TTCTCTGCCTTGCCTACGAC
	R	GCAGAGCTGACAAACAGACG
CXCL2	F	CTCAAGAATGGGCAGAAAGC
	R	CTTCAGGAACAGCCACCAAT
CXCL3	F	TGTGAATGTAAGGTCCCCCG
	R	TTGGTGCTCCCCTTGTTCAG
CSF2	F	GGGAGCATGTGAATGCCATC
	R	GGCTCCTGGAGGTCAAACAT
MAGE-A6	F	CAGAGGAGTCAGCACTGCAA
	R	GACTCTGGGGAGGATCTGGT
DST	F	TGCCAACAGAGTTCAGAGGG
	R	GCTGGCGTAAAAGGTTCTCAC
IL-8	F	GGTGCAGTTTTGCCAAGGAG
	R	TTCCTTGGGGTCCAGACAGA

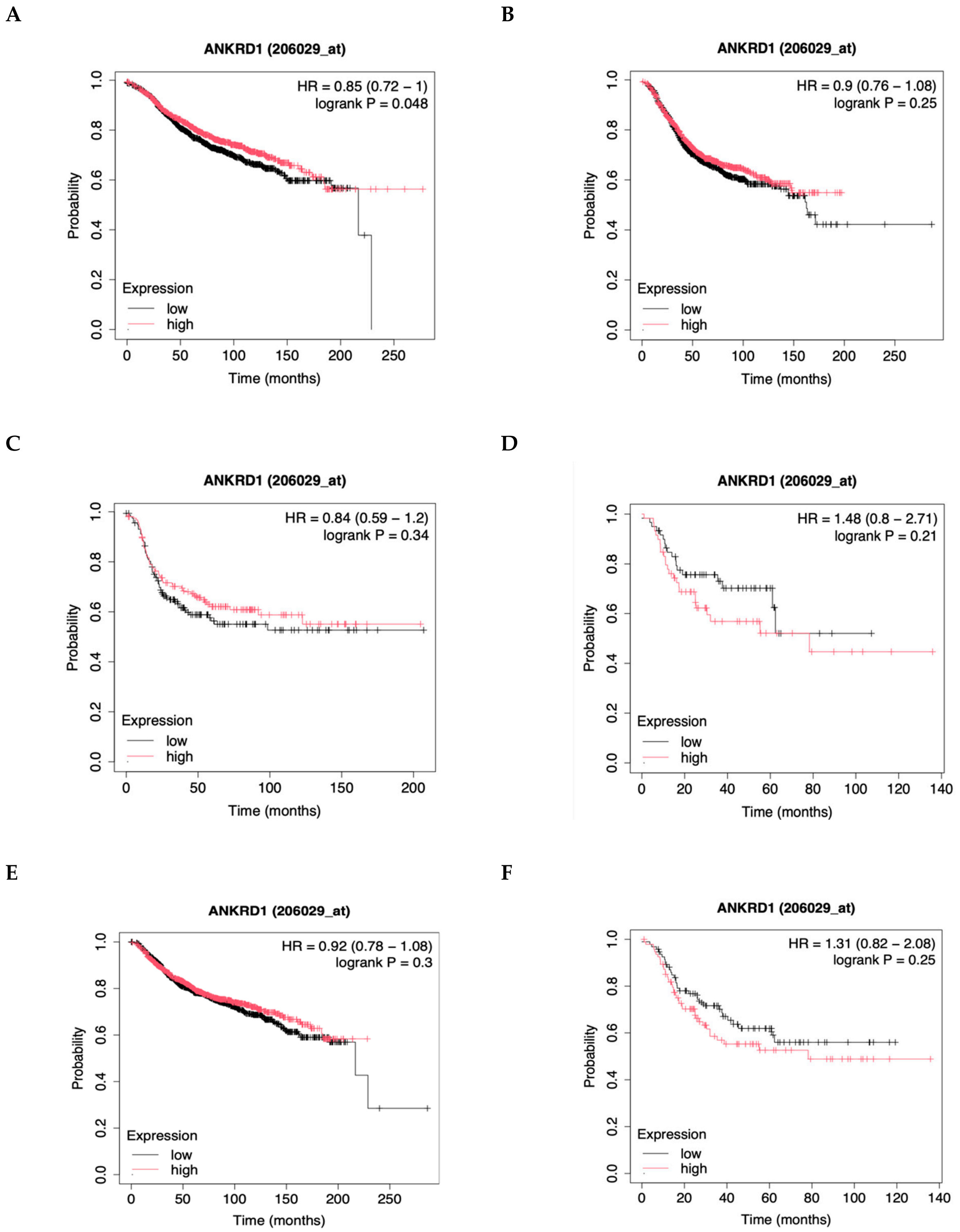

Figure S1. "ANKRD1" gene in TCGA on the database in breast cancer using Kaplan-Meier survival analysis (mRNA gene chip). **(A)** Survival rate of luminal A subtype. **(B)** Survival rate of luminal B subtype. **(C)** Survival rate of HER2 positive subtype. **(D)** Survival rate of triple negative subtype. **(E)** Survival rate of lymph node negative. **(F)** Survival rate of lymph node positive. ns > 0.01.

A

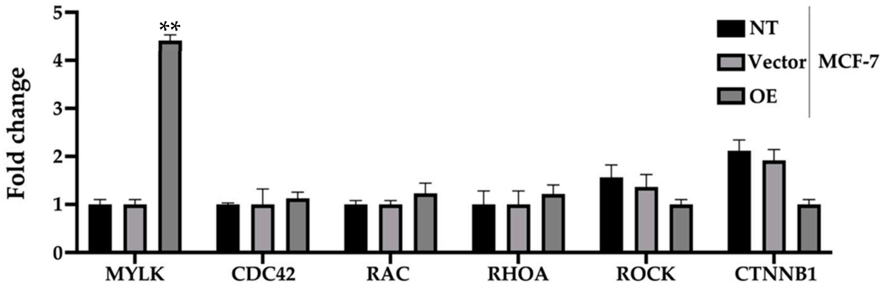

B

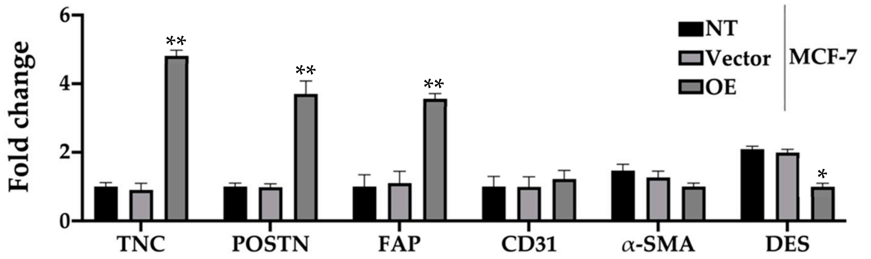

Figure S2. Comparison of migration and adhesion and cancer associated fibroblasts markers. **(A)** Real time PCR results showing fold change of migration and adhesion markers. The data shows upregulation of MYLK and downregulation of CTNNB1 in OE cells compared to NT and Vector. **(B)** Real time PCR results showing fold change of cancer associated fibroblasts (CAFs) markers. The data shows upregulation of TNC, POSTN, FAP and downregulation of DES in OE cells compared to NT and Vector. NT: MCF-7 parental, Vector: MCF-7-emtpy vector and OE: MCF-7-ANKRD1 overexpression. All experiments were performed in triplicate. **≤0.001 and *≤0.05.

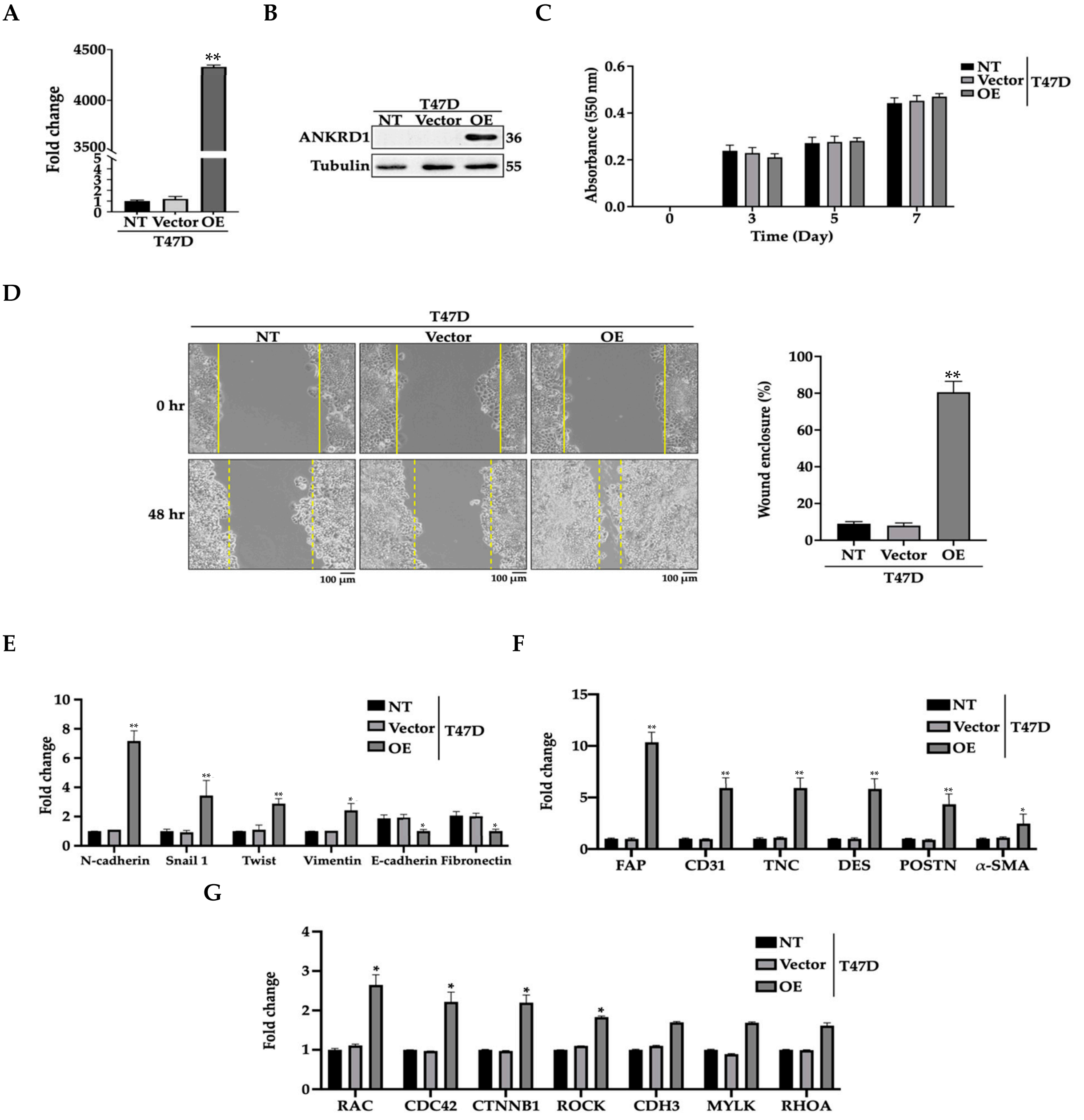

Figure S3. Overexpression of ANKRD1 induces cell migration and invasion in T47D, a non-metastatic breast cancer **(A-B)** Results of real time PCR and Western blot assay showing the gene and protein expression in the ANKRD1 overexpressed cell lines (T47D-ANKRD1 OE, OE) **(C)** Cell proliferation of ANKRD1 overexpressing cells compared to vector and normal T47D was determined by EZ-Cytox assay. **(D)** Representative images depicting wound healing quantified as a percentage of the healed wound area and analyzed by Image JS. **(E-G)** Real time PCR showing the gene expression involved in EMT, migration-adhesion and CAFs. OE cells showed reduced expression of E-cadherin and increased expression of N-cadherin, Snail1, Twist, FAP, CD31, TNC, DES, POSTN, α-SMA, RAC, CDC42, CTNNB1, ROCK,CDH3, MYLK and RHOA compared to Vector. NT: T47D parental, Vector: T47D-emtpy vector and OE: T47D-ANKRD1 overexpression. All experiments were performed in triplicate. **≤0.001 and *≤0.05.

A

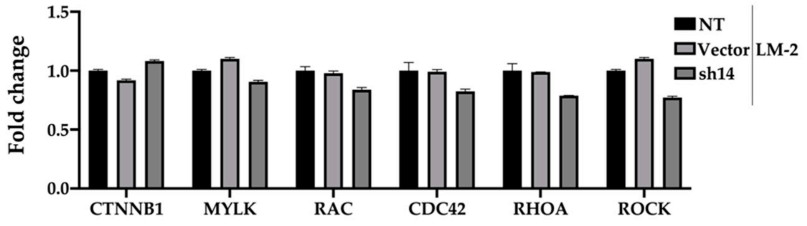

B

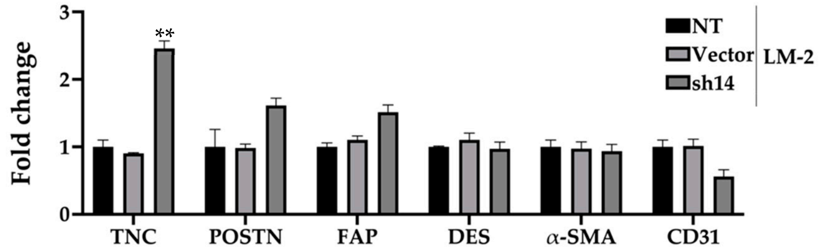

Figure S4. Comparison of migration and adhesion and cancer associated fibroblasts markers **(A)** Real time PCR results showing fold change of migration and adhesion markers. The data shows downregulation of RHOA and ROCK in sh14 cells compared to NT and Vector. **(B)** Real time PCR results showing fold change of cancer associated fibroblasts (CAFs) markers. The data shows upregulation of TNC, POSTN and FAP and downregulation of CD31 in sh14 cells compared to NT and Vector.NT: LM-2 parental, Vector: LM-2 with empty vector and sh14: LM-2-ANKRD1 knockdown with sh14. All experiments were performed in triplicate. **≤0.001 and *≤0.05.

A B

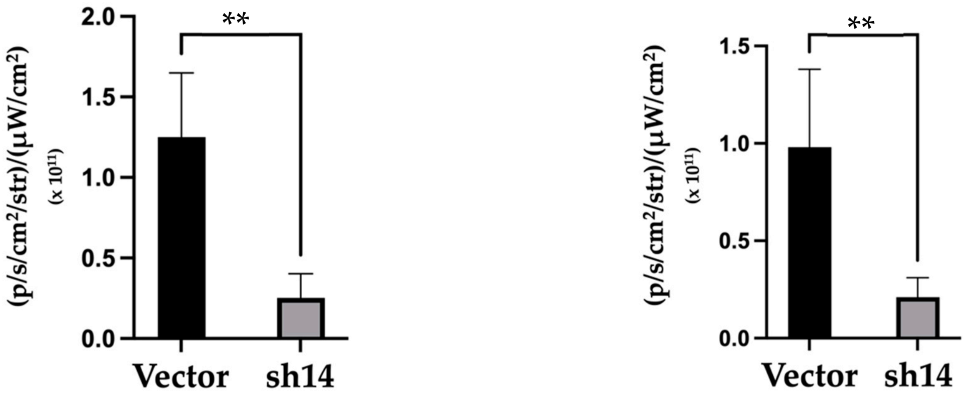

Figure S5. Comparison of the radiant efficacy of different organs at day 28 *in vivo* metastasis models **(A)** Radiant efficacy of lung. **(B)** Radiant efficacy of liver. **≤0.001 and *≤0.05.

A

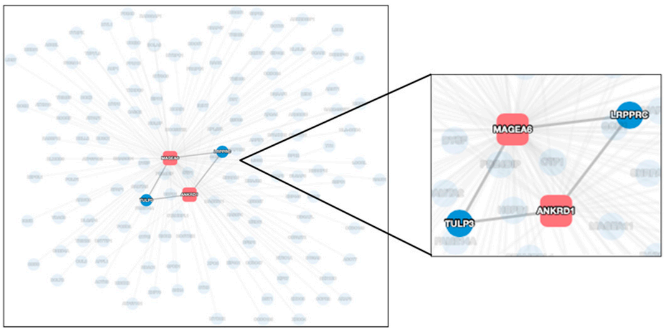

Figure S6. Protein-protein interaction **(A)** Protein interaction between ANKRD1 and MAGE-A6 using http://omics.bjcancer.org.

Table S2. List of siRNAs used in the study.

siRNA name	Duplex Sequence (5′-3′)	
MAGE-A6	Duplex 1	GAUGGUUGAAUGAGCGUCAdTdT
	Duplex 2	GGUAAAGAUCAGUGGAGGAdTdT
NF-κB #1	Sense	UUUCUCAUCCCAUCUUUGAUU
	Anti-Sense	UCAAAGAUGGGAUGAGAAAUU
NF-κB #2	Sense	GUGACAAGGUGCAGAAAGAUU
	Anti-Sense	UCUUUCUGCACCUUGUCACUU
NF-κB #3	Sense	GACAUUGAGGUGUAUUUCAUU
	Anti-Sense	UGAAAUACACCUCAAUGUCUU
NF-κB #4	Sense	ACAUAUGAGACCUUCAAGAUU
	Anti-Sense	UCUUGAAGGUCUCAUAUGUUU
Negative control	Sense	CCUCGUGCCGUUCCAUCAGGUAGUU
	Anti-Sense	CUACCUGAUGGAACGGCACGAGGUU

A

Uncategorized

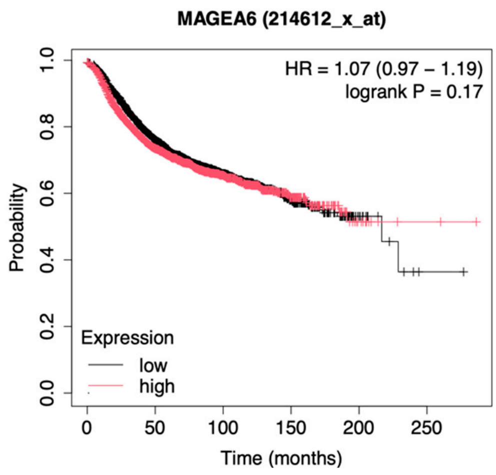

B

All three negative (TBNC)

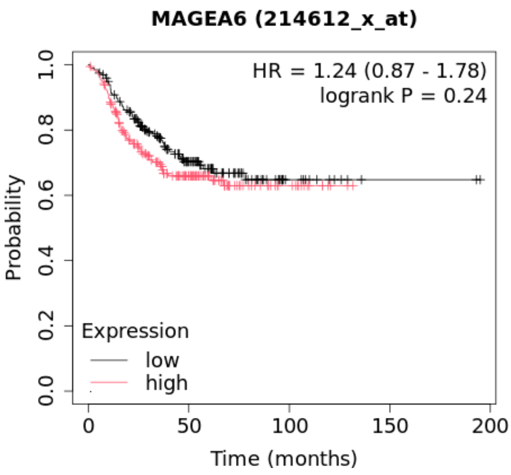

C

Lymph node negative

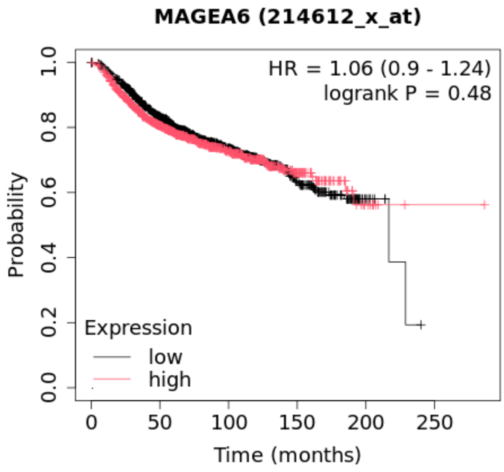

D

Lymph node positive

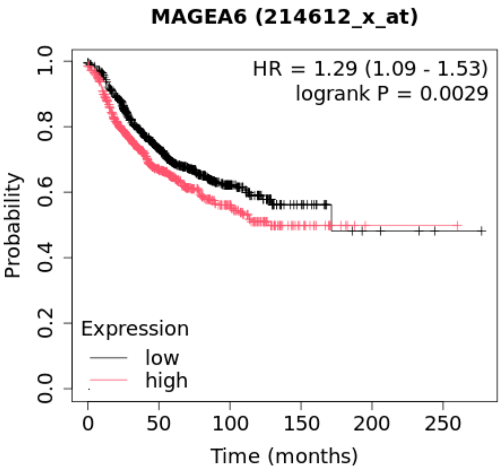

Figure S7. "MAGEA6" gene in TCGA on the database in breast cancer using Kaplan-Meier survival analysis (mRNA gene chip) **(A)-(B)** Comparison of Kaplan–Meier survival analysis between receptor types of breast cancer. **(C)-(D)** Comparison of Kaplan–Meier survival analysis lymph node system of breast cancer. ns > 0.01.
